# First-line opioid agonist treatment as prevention against assisting others in initiating injection drug use: A longitudinal cohort study of people who inject drugs in Vancouver, Canada

**DOI:** 10.1016/j.dadr.2023.100168

**Published:** 2023-05-25

**Authors:** Zachary Bouck, Andrea C. Tricco, Laura C. Rosella, Hailey R. Banack, Matthew P. Fox, Robert W. Platt, M-J Milloy, Kora DeBeck, Kanna Hayashi, Dan Werb

**Affiliations:** a Epidemiology Division, Dalla Lana School of Public Health, University of Toronto, Toronto, ON, Canada; b Centre on Drug Policy Evaluation, St. Michael's Hospital, Unity Health Toronto, Toronto, ON, Canada; c Knowledge Translation Program, St. Michael's Hospital, Unity Health Toronto, Toronto, ON, Canada; d Institute for Health Policy, Management and Evaluation, University of Toronto, Toronto, ON, Canada; e ICES, Toronto, ON, Canada; f Department of Epidemiology, Boston University School of Public Health, Boston, MA, United States; g Department of Global Health, Boston University School of Public Health, Boston, MA, United States; h Department of Epidemiology, Biostatistics, and Occupational Health, McGill University, Montréal, QC, Canada; i Department of Pediatrics, McGill University, Montréal, QC, Canada; j British Columbia Centre for Substance Use, Vancouver, BC, Canada; k Department of Medicine, University of British Columbia, Vancouver, BC, Canada; l Faculty of Health Sciences, Simon Fraser University, Burnaby, BC, Canada; m Division of Infectious Diseases and Global Public Health, University of California San Diego, La Jolla, CA, United States

**Keywords:** Injection drug use, Injection initiation, Opioid agonist treatment, Opioid agonist therapy

## Abstract

**Background:**

Among people who inject drugs, frequent injecting and experiencing withdrawal are associated with facilitating others’ first injections. As these factors may reflect an underlying substance use disorder, we investigated whether first-line oral opioid agonist treatment (OAT; methadone or buprenorphine/naloxone) reduces the likelihood that people who inject drugs help others initiate injecting.

**Methods:**

We used questionnaire data from semi-annual visits between December 2014–May 2018 on 334 people who inject drugs with frequent non-medical opioid use in Vancouver, Canada. We estimated the effect of current first-line OAT on subsequent injection initiation assistance provision (i.e., helped someone initiate injecting in the following six months) using inverse-probability-weighted estimation of repeated measures marginal structural models to reduce confounding and informative censoring by time-fixed and time-varying covariates.

**Results:**

By follow-up visit, 54–64% of participants reported current first-line OAT whereas 3.4–6.9% provided subsequent injection initiation assistance. Per the primary weighted estimate (n = 1114 person-visits), participants currently on first-line OAT (versus no OAT) were 50% less likely, on average, to subsequently help someone initiate injecting (relative risk [RR]=0.50, 95% CI=0.23–1.11). First-line OAT was associated with reduced risk of subsequent injection initiation assistance provision in participants who, at baseline, injected opioids less than daily (RR=0.15, 95% CI=0.05–0.44) but not in those who injected opioids daily (RR=0.86, 95% CI=0.35–2.11).

**Conclusions:**

First-line OAT seemingly reduces the short-term likelihood that people who inject drugs facilitate first injections. However, the extent of this potential effect remains uncertain due to imprecise estimation and observed heterogeneity by baseline opioid injecting frequency.

## Highlights

• Most first-time injections involve assistance from people who inject drugs.
• Having an untreated opioid use disorder may increase risk of providing assistance.
• First-line opioid agonist treatment could therefore reduce assistance provision.
• We found evidence to support this presumed protective treatment effect.
• Effect magnitude uncertain due to imprecise estimation and observed heterogeneity.

## 1 Introduction

People who inject drugs are at increased risk of overdose, HIV, hepatitis C, and severe bacterial infections (Backmund et al., 2005; Brugal et al., 2002; Darke, 2003; Degenhardt et al., 2017, 2016; Garfein et al., 1996; Noroozi et al., 2020; Tagliaro et al., 1998; van Haastrecht et al., 1996), especially within the first few years after they begin injecting (Garfein et al., 1998; Goldsamt et al., 2010). Drug injecting is generally learned by injection-naïve people who use drugs through observing and interacting with people who inject drugs in shared social circles and drug use environments (Harocopos et al., 2009; Rhodes et al., 2011; Strike et al., 2014; Vashishtha et al., 2017; Werb et al., 2018). By engaging in injection-promoting behaviours (including injecting in front of injection-naïve individuals and speaking positively about injecting), people who inject drugs can normalize injecting and increase its appeal to others (Crofts et al., 1996; Des Jarlais et al., 2021; Frajzyngier et al., 2007; Harocopos et al., 2009; Khobzi et al., 2009; Small et al., 2009; Strike et al., 2014; Witteveen et al., 2006). Repeated exposure to injection practices combined with additional factors, such as increased drug tolerance and dependence, may influence injection-naïve people who use drugs to consider injecting and seek help initiating (Bryant and Treloar, 2007; Crofts et al., 1996; Strike et al., 2014; Witteveen et al., 2006). People who inject drugs may further facilitate others’ transitions to injecting by providing assistance with first-time injections, either directly (i.e., they inject the initiate) or indirectly (i.e., they explain, describe, or demonstrate how to inject to an initiate who then injects themselves for their first time) (Des Jarlais et al., 2019; Des Jarlais et al., 2021; Gicquelais et al., 2020; Uusküla et al., 2018). In fact, between 76 and 90% of sampled people who inject drugs in Canada and the US report that they received assistance from an experienced person who injects drugs in initiating injecting, with most (range=55–86%) having been directly injected by someone else (Gicquelais et al., 2020).

Both frequent and public injecting have been associated with engaging in injection-promoting behaviours, being asked to help someone inject, and ultimately providing injection initiation assistance among people who inject drugs (Bluthenthal et al., 2015a, 2015b; Melo et al., 2018; Mittal et al., 2019b; Navarro et al., 2019; Rotondi et al., 2014; White et al., 2020). Injecting frequently in public spaces can increase the visibility of one's injecting practices to injection-naïve individuals, potentially resulting in more requests and opportunities to assist with first injections (Des Jarlais et al., 2021; Melo et al., 2018; Mittal et al., 2019b; Navarro et al., 2019; White et al., 2020). Though people who inject drugs consistently describe a group ethic or “moral code” against helping others initiate injecting due to injection-related harms, some report reluctantly providing such assistance in exchange for drugs (or money to obtain drugs) to meet their own drug use needs, including avoiding or mitigating withdrawal symptoms (Goldsamt et al., 2010; Guise et al., 2018; Guise et al., 2017; Kolla et al., 2015; Mittal et al., 2019a; Olding et al., 2019; Rhodes et al., 2011; Simpson et al., 2020). This commonly expressed motivation may further explain why frequent injecting, which is correlated with increased drug tolerance and dependence, is positively associated with providing injection initiation assistance (Rotondi et al., 2014; Simpson et al., 2020; Werb et al., 2018).

The *PReventing Injecting by Modifying Existing Responses* (PRIMER) study is a consortium of existing cohorts across North America (including Vancouver, Canada; San Diego, US; and Tijuana, Mexico) that aims to identify interventions that might reduce the likelihood that people who inject drugs provide injection initiation assistance (Werb et al., 2016). Initial PRIMER evidence suggests that opioid agonist treatment (OAT) with methadone or buprenorphine/naloxone—the recommended first-line oral pharmacotherapies for opioid use disorder in Canada and many other countries (Bruneau et al., 2018; Canadian Research Initiative in Substance Misuse (CRISM), 2018; Centre for Addiction and Mental Health (CAMH), 2021; Dunlap and Cifu, 2016; Volkow et al., 2014)—might offer such a benefit (Marks et al., 2019, 2021b; Meyers-Pantele et al., 2022; Mittal et al., 2017, 2019b). Specifically, prior cross-sectional studies of Vancouver-based people who inject drugs found that participants enrolled in first-line OAT within the past six months had reduced odds of helping someone initiate injecting over the same period (Marks et al., 2019, 2021b; Mittal et al., 2019b). These observed associations are presumably owed to the known effectiveness of methadone and buprenorphine/naloxone in alleviating opioid cravings and preventing withdrawal symptoms in treated people who inject drugs, leading to reductions in non-medical opioid (and overall) injecting (Gowing et al., 2011; Karki et al., 2016; Mattick et al., 2009; Mittal et al., 2019b; Volkow et al., 2014). Due to these primary benefits, people who inject drugs on first-line OAT may be less likely to expose others to their injecting practices (because of reductions in overall and public injecting) versus untreated individuals, resulting in fewer requests from injection-naïve individuals to assist them in initiating injecting (Des Jarlais et al., 2021; Kolla et al., 2015; Mittal et al., 2019b). Furthermore, by preventing opioid cravings and withdrawal, people who inject drugs on a stable OAT dose may be less vulnerable to accepting requests to provide injection initiation assistance in exchange for drugs or money to procure drugs (Des Jarlais et al., 2021; Kolla et al., 2015; Mittal et al., 2019b). As an estimated 73% of people who inject drugs in North America primarily inject opioids but only 20% are on OAT (Degenhardt et al., 2017; Larney et al., 2017), these preliminary findings suggest that increasing OAT coverage in this population could substantially limit injection drug use initiation, in addition to reducing injecting and related harms in treated individuals (Marks et al., 2021b, 2019; Meyers-Pantele et al., 2021; Mittal et al., 2019b).

Though a secondary preventative effect of first-line OAT on injection initiation assistance provision in people who inject drugs seems plausible, further investigation is warranted due to limitations of the initial studies assessing this relationship (Marks et al., 2019, 2021b; Meyers-Pantele et al., 2022; Mittal et al., 2017, 2019b). First, prior studies were not restricted to individuals with a suspected opioid use disorder, meaning OAT-ineligible participants were likely included (Marks et al., 2019, 2021b; Meyers-Pantele et al., 2022; Mittal et al., 2017, 2019b). Second, the initial evidence is from cross-sectional analyses involving contemporaneous confounder, exposure, and outcome measures (Marks et al., 2019, 2021b; Meyers-Pantele et al., 2022; Mittal et al., 2017, 2019b). Without clarity in the temporal sequence of these measures, there are other plausible explanations for previously observed associations beyond treatment preventing injection initiation assistance provision, including reverse causality (Marks et al., 2019, 2021b; Meyers-Pantele et al., 2022; Mittal et al., 2017, 2019b). Third, earlier studies used participant-reported measures of both OAT and injection initiation assistance provision but did not account for misclassification bias (e.g., inaccurate recall and socially desirable responding) in these measures (Bouck et al., 2022).

In this study, we aimed to estimate the short-term effect of first-line OAT on injection initiation assistance provision within a cohort of people who inject drugs in Vancouver, Canada, while addressing the aforementioned issues by restricting to participants with suspected opioid use disorder; using longitudinal data to establish temporality between confounder, exposure, and outcome measures; and adjusting for misclassification in participant-reported treatment and outcome measures. We hypothesized that participants currently on first-line OAT with methadone or buprenorphine/naloxone would be less likely, on average, to help someone initiate injecting in the next six months compared to participants not on any medication for opioid use disorder.

## 2 Methods

### 2.1 Data sources

We used questionnaire data collected from December 2014-May 2018 on participants from three prospective, open, and linked cohorts in Vancouver, British Columbia: the *Vancouver Injection Drug Users Study* (VIDUS); the *AIDS Care Cohort to Evaluate exposure to Survival Services* (ACCESS) study; and the *At-Risk Youth Study* (ARYS). This period corresponds with VIDUS/ACCESS/ARYS survey cycles 18–24 and overlaps with prior studies of OAT and injection initiation assistance provision in Vancouver (Marks et al., 2019, 2021b; Mittal et al., 2019b). Beyond living in the Vancouver area and providing informed consent, inclusion criteria were: for VIDUS, that participants be ≥18 years old, HIV-negative, and report injecting drugs in the past month; for ACCESS, that participants be ≥18 years old, HIV-positive, and report illicit drug use in the past month; and for ARYS, that participants be 14–26 years old, “street-involved” (recently homeless or used street youth services), and report illicit drug use in the past month (Werb et al., 2016). At enrollment and semi-annual visits thereafter, VIDUS/ACCESS/ARYS participants complete harmonized interviewer-administered questionnaires that collect information on their sociodemographic characteristics, drug use and related behaviours, and history of treatment for substance use disorders (Werb et al., 2016). Under PRIMER, survey items asking about participants’ experiences helping others initiate injecting were added to VIDUS/ACCESS/ARYS questionnaires in December 2014 (Werb et al., 2016). This study was approved by the University of California San Diego Human Research Protection Program, the University of British Columbia/Providence Health Care and the University of Toronto Research Ethics Boards (#27433).

### 2.2 Participants

We included VIDUS/ACCESS/ARYS participants who attended a visit in each of the first two survey cycles involving PRIMER questions, i.e., cycles 18 (December 2014-May 2015) and 19 (June 2015-November 2015). Hereafter, we refer to the first and second of these visits by date as a participant's ‘look-back’ and ‘baseline’ visits, respectively. We then restricted to those participants who, at their baseline visit, reported ever injecting drugs and frequent (i.e., ≥weekly) non-medical opioid use via injection and/or non-injection over the past six months. These criteria were intended to select participants with injecting experience (at-risk for providing injection initiation assistance) and a probable opioid use disorder (presumably OAT-eligible) (Socías et al., 2018). To facilitate comparisons between individuals on first-line OAT versus no OAT, we excluded participants who reported OAT with an alternative opioid agonist medication including slow-release oral morphine, hydromorphone (oral or injectable), or injectable diacetylmorphine at their look-back visit or within the six months preceding, and inclusive of, their baseline visit (**Appendix 1**) (Oviedo-Joekes et al., 2016). Last, we excluded participants with missing baseline exposure, covariate, or outcome data (see *Measures* section).

Each participant contributed up to 5 follow-up visits from December 2015-May 2018, beginning with their first semi-annual visit after baseline and ending with the last visit they attended before (1) their first missed outcome measurement (due to missed visit, invalid or non-response), (2) their first visit where they reported recent OAT with an alternative opioid agonist medication (**Appendix 1**), or (3) June 2018 (administrative censoring), whichever came first.

### 2.3 Measures

Current first-line OAT was assessed at all visits and defined as a “yes” response to the question: “In the last six months, have you been in any kind of alcohol or drug treatment?” with specification of enrollment, as of the interview, in OAT with methadone or buprenorphine/naloxone (Bouck et al., 2022; Socías et al., 2019; Socías et al., 2017). Recent injection initiation assistance provision was measured at all visits and defined as a “yes” response to the question: “In the last six months, have you helped anybody inject who had never injected before?” (Werb et al., 2016).

**Fig. 1:**
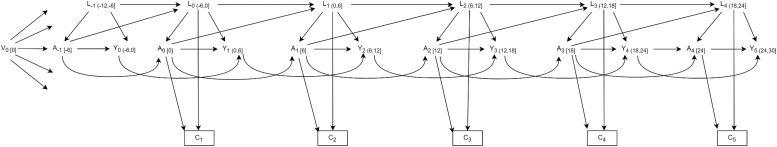
Assumed relationships among study measures. *Notes: t* denotes semi-annual study visit, where *t*=(−1,0,1,2,3,4,5) with *t=*−1 indicating a participant's look-back visit six months before their baseline visit (*t* = 0); V_0_ denotes time-independent covariates measured at baseline (*t* = 0); A*_t_* denotes exposure (current opioid agonist treatment) at visit *t*; L*_t_* denotes time-varying covariates measured at visit *t*; Y*_t_* denotes outcome measured at visit *t*; and C*_t_* denotes whether a participant was censored as of visit *t* (the boxes around C*_t_* reflect that our analyses were restricted to uncensored person-visit observations, i.e., C*_t_*=0). Time-independent baseline covariates (V_0_) were assumed to affect all other measured variables. The subscript number(s) in parentheses provided beside each variable (or set of variables) denotes the period or point of assessment (in months from baseline) captured by these variables (e.g., Y_1 (0,6]_ is the outcome measured at *t* = 1 [first follow-up visit] and it captures whether participants provided injection initiation assistance in the six months between baseline (exclusive) and their first follow-up visit [inclusive]).

We additionally measured time-independent and time-varying covariates that were presumed to confound the relationship between first-line OAT and subsequent injection initiation assistance provision and influence the likelihood of premature censoring during follow-up (Fig. 1). Measured time-independent baseline covariates included: age (in years; treated as continuous), self-identified gender (cisgender man; yes/no), and cohort (VIDUS, ACCESS, or ARYS) (Ben Hamida et al., 2018; Kerr et al., 2005; Marks et al., 2019, 2021b; Melo et al., 2018; Meyers-Pantele et al., 2021). Measured time-varying covariates, which were assessed at baseline and follow-up visits, included: recent homelessness; recent incarceration (jailed, imprisoned, or detained minimally overnight); indicators of recent income from the following sources (note: multiple sources could be selected): paid legal work, social assistance, street-based activities (i.e., panhandling, squeegeeing, or recycling), sex work, or illegal activities (i.e., drug dealing, theft, or other criminal activity); recent non-fatal overdose; recent frequency (daily, weekly, less-than-weekly, or none) of both opioid and non-opioid (e.g., cocaine, crystal methamphetamine, benzodiazepines) injection drug use; and recent public injection (Bouck et al., 2020; Bowles et al., 2021; Kerr et al., 2005; Luongo et al., 2017; Marks et al., 2021a; Mittal et al., 2019b; Navarro et al., 2019; White et al., 2020). Covariates qualified as ‘recent’ reflect behaviours, experiences, and/or activities over the past six months. If a time-varying covariate value was missing (true for <0.5% of uncensored person-visit observations), we carried the last observed value forward.

### 2.4 Statistical analysis

To evaluate our hypothesis and account for repeated outcome measurements, we fit a weighted log-binomial model using generalized estimating equations with an unstructured working correlation matrix for within-participant responses (Ballinger, 2004; Hernán et al., 2002; Hernán and Robins, 2020):

(model 1) $$\begin{array}{ccc} & & \text{log}\left(\text{Pr}\left[Y_{\mathrm{it}}=1|A_{\mathrm{it}-1},\;A_{\mathrm{it}-2},\;{\bar{C}}_{\mathrm{it}}=\bar{0}\right]\right)\\ & & =\beta_{0t}+\beta_{1}A_{\mathrm{it}-1}+\beta_{2}A_{\mathrm{it}-2}, \end{array}$$

where i indexes participants; *t* denotes visit (range=1–5); $Y_{it}$ indicates whether participant i reported recent injection initiation assistance provision (1=yes, 0=no) at visit *t*; $A_{it-1}$ and $A_{it-2}\;$ indicate whether participant i reported current first-line OAT (1=yes, 0=no) at visit *t*-1 and *t*-2 respectively, with $A_{it-2}$ included since it was used to stabilize the weights (Cole and Hernán, 2008; Hernán et al., 2002; Keogh et al., 2018; Robins et al., 2000); ${\bar{C}}_{it}=\bar{0}$ reflects that the analysis was restricted to uncensored person-visit observations (Moodie et al., 2008); $\beta_{0t}\;$ represents visit-specific intercepts; and $\beta_{1}$ expresses the focal exposure-outcome association on the log probability scale. Assuming exchangeability (no unmeasured confounding and ignorable censoring), consistency (treatment levels under comparison correspond with well-defined interventions and measured levels), positivity (participants have non-zero probabilities of being treated or untreated conditional on their measured treatment and covariate histories), and no weight or outcome model misspecifications, the model 1 coefficient $\beta_{1}$ is an unbiased estimate of the causal parameter $\;\gamma_{1}$ from the following repeated measures marginal structural model (Cole and Hernán, 2008; Hernán et al., 2002; Hernán and Robins, 2020; Robins et al., 2000):

(model 2) $$\text{log}\left(\text{Pr}\left[Y_{\mathrm{it}}^{a_{\mathrm{it}-1}}=1|a_{\mathrm{it}-2}\right]\right)=\gamma_{0t}+\gamma_{1}a_{\mathrm{it}-1}+\gamma_{2}a_{\mathrm{it}-2},$$

where $Y_{it}^{a_{it-1}}$ represents a participant's potential outcome at visit *t* had their treatment at visit *t*-1 been set, possibly contrary to their observed exposure, to $a_{it-1}$ (current OAT: 1=yes, 0=no) and $\exp(\gamma_{1})\;$ is the treatment effect of interest—expressed as a relative risk (RR)—which compares the risk of recent injection initiation assistance provision at visit *t* had everyone (all uncensored participants by visit *t*) been on first-line OAT at visit *t*-1 versus had everyone not been on any medication for opioid use disorder at visit *t*-1, conditional on everyone having the same treatment status at visit *t*-2 (Hernán et al., 2001; Hernán and Robins, 2020; Keogh et al., 2018; Robins et al., 2000). A 95% confidence interval (CI) was calculated using a robust, sandwich-type variance estimator (Hernán et al., 2002). In anticipation of a rare outcome, we analyzed repeated outcome measures to increase precision in estimation (VanderWeele et al., 2011).

To account for confounding and non-ignorable censoring (i.e., differential loss-to-follow-up) by measured time-independent and time-varying covariates, we fit model 1 using inverse probability-of-treatment-and-censoring (IPTC) weights (Cole and Hernán, 2008). Informally, the IPTC weight for participant *i* at visit *t* was inversely proportional to that participant's probability of remaining uncensored by visit *t* and receiving their reported treatment at visit *t*-1 conditional on their observed treatment and covariate histories (Cole and Hernán, 2008). Formally, the IPTC weight for the it^th^ person-visit contributing to model 1 was calculated as the product of their stabilized inverse probability-of-treatment (IPT) weight, ${\text{SW}}_{it}^{A},\;$ and inverse-probability-of-censoring (IPC) weight, ${\text{SW}}_{it}^{C}$:

$${\text{SW}}_{\mathrm{it}}^{A}=\prod_{k=1}^{t}\frac{\mathrm{Pr}\left[A_{\mathrm{ik}-1}|{\bar{A}}_{\mathrm{ik}-2,}{\bar{C}}_{\mathrm{ik}-1}=\bar{0}\right]}{\mathrm{Pr}\left[A_{\mathrm{ik}-1}|{\bar{A}}_{\mathrm{ik}-2},\;{\bar{C}}_{\mathrm{ik}-1}=\bar{0},\;V_{i0},\;{\bar{L}}_{\mathrm{ik}-1}\right]},$$

and

$${\text{SW}}_{\mathrm{it}}^{C}=\prod_{k=1}^{t}\;\frac{\mathrm{Pr}\left[C_{\mathrm{ik}}=0|{\bar{C}}_{\mathrm{ik}-1}=\bar{0},\;\;{\bar{A}}_{\mathrm{ik}-1}\right]}{\mathrm{Pr}\left[C_{\mathrm{ik}}=0|{\bar{C}}_{\mathrm{ik}-1}=\bar{0},\;\;{\bar{A}}_{\mathrm{ik}-1},\;\;V_{i0},\;{\bar{L}}_{\mathrm{ik}-1}\right]},$$

where *t*=(1–5); $C_{ik}$ flags whether a participant was censored by visit *k* (1=yes, 0=no) (Moodie et al., 2008); $V_{i0}$ represents the vector of measured time-independent baseline covariates; $L_{ik-1}$ represents the vector of measured time-varying covariates at visit *k*-1; and an overbar signifies the observed history of a time-varying variable, e.g., ${\bar{L}}_{ik-1}$=($L_{i0},\;\;\cdots ,\;L_{\mathrm{ik}-1}$). Weight components were estimated using pooled logistic regression (**Appendix 2**). Application of the IPTC weights creates a pseudo-population where treatment selection and censoring are statistically independent of measured time-independent and time-varying covariates featured exclusively in the denominator of the weights (Cole and Hernán, 2008; Hernán et al., 2002; Hernán and Robins, 2020; Howe et al., 2016). In other words, the IPTC weights remove the arrows in Fig. 1 from the $V_{0}$ and $L_{t-1}\;$ variables into $A_{t-1}$ and $C_{t}$ where *t*=(1–5) (**Appendix 2**). With a rare outcome and more common exposure, weighting to control for confounding facilitates model convergence and reduces overfitting versus traditional covariate adjustment (Greenland et al., 2016; Richardson et al., 2015; Schuster et al., 2016). While our eligibility criteria were intended to select OAT-eligible participants, we visually inspected estimated weight distributions over time for means far from one or extreme values, which could indicate positivity violations or weight model misspecifications (Cole and Hernán, 2008; Hernán and Robins, 2020). To explore the influence of measured confounding and non-ignorable censoring, we alternatively fit model 1 using IPT weights, IPC weights, and no weights for comparison with the IPTC-weighted estimate.

### 2.5 Sensitivity analysis

We assessed the joint impact of unmeasured confounding and non-ignorable censoring by prior outcome response ($Y_{it-1}$) by adding this variable to the treatment and censoring weight denominators and re-fitting model 1 with the updated IPTC weights.

We conducted quantitative bias analyses to explore the impact of self-reported treatment misclassification bias on the direction and magnitude of our effect estimate. First, we assumed self-reported exposure (current OAT at visit *t*-1; $A_{it-1}$) misclassification was non-differential by outcome ($Y_{it}$) and assigned beta distributions for sensitivity (α=116.6, β=22.2; mean=84%, standard deviation [SD]=3.1%) and specificity (α=245.1, β=36.6; mean=87%, SD=2.0%) based on validation data from a sample of people who inject drugs in Toronto, Ontario (**Appendix 3**) (Bouck et al., 2022). Using a method described by Fox et al. and adapting open-access code (**Appendix 4**), we: (1) randomly sampled sensitivity and specificity (bias parameters) from the beta distributions describing our uncertainty in their values; (2) used the selected parameter values to adjust person-visit observations for exposure misclassification; (3) re-fit model 1 (unweighted) in the bias-adjusted dataset; and (4) repeated steps (1)-(3) for 10,000 iterations (**Appendix Figure 1**) (Fox et al., 2022; Fox et al. 2021). The median bias-adjusted RR was reported with 95% simulation intervals (SI) incorporating both systematic error (uncertainty in the bias parameter distributions) and random error (Fox et al., 2021). Second, we followed the same steps as the preceding analysis but additionally updated the value of the treatment covariate (current OAT at visit *t*-2; $A_{it-2})$ for person-visit observations where *t*=(1–5) based on the lagged value of a participants’ bias-corrected exposure ($A_{it-1}$) within the same simulated dataset. We reported the median RR with 95% SI after adjusting for joint exposure and covariate misclassification (**Appendix 4**).

We followed the same step-by-step approach as the exposure-only misclassification bias analysis to adjust for suspected outcome underreporting (**Appendix 5**) (Fox et al., 2022; Fox et al. 2021). Without validation data, we assumed that outcome ($Y_{it}$) misclassification was differential by reported exposure $\left(A_{it-1}\right)$, with modestly-improved recall among exposed (sensitivity∼beta[α=33.7, β=9.5; mean=78%, SD=6.2%] and specificity∼beta[α=788.5, β=0.3; mean=99%, SD=0.1%]) versus unexposed participants (sensitivity∼beta[α=40.6, β=16.6; mean=71%, SD=6.0%] and specificity∼beta[α=558.4, β=0.4; mean=99%, SD=1.1%]) (**Appendix Figure 2**). All bias analysis results (median RR with 95% SI) were compared with the conventional, unweighted estimate.

### 2.6 Post hoc analysis

As individuals with severe opioid use disorder characterized by high-frequency opioid injecting may not adequately benefit from first-line OAT for various reasons (e.g., cannot reach therapeutic dose or persistent cravings) (Fairbairn et al., 2019), we investigated whether the short-term effect of first-line OAT on injection initiation assistance provision differs by baseline opioid injecting frequency. Specifically, we added terms to the primary IPTC-weighted outcome model for daily baseline opioid injecting ($V_{i0}^{*}$: 1=yes, 0=no)—intended as an indicator of severe, potentially treatment-refractory opioid use disorder—and its product with current OAT at visit *t*-1 (Cole and Hernán, 2008):

$$\begin{array}{ccc} & & \text{log}\left(\text{Pr}\left[Y_{\mathrm{it}}=1|A_{\mathrm{it}-1},\;A_{\mathrm{it}-2},\;V_{i0}^{*},\;\;{\bar{C}}_{\mathrm{it}}=\bar{0}\right]\right)=\\ & & \beta_{0t}+\beta_{1}A_{\mathrm{it}-1}+\beta_{2}A_{\mathrm{it}-2}+\beta_{3}V_{i0}^{*}+\beta_{4}\left(A_{\mathrm{it}-1}*V_{i0}^{*}\right) \end{array}$$

All analyses were conducted in SAS V9.4 (SAS Institute Inc.; Cary, NC).

## 3 Results

### 3.1 Baseline characteristics

**Fig. 2:**
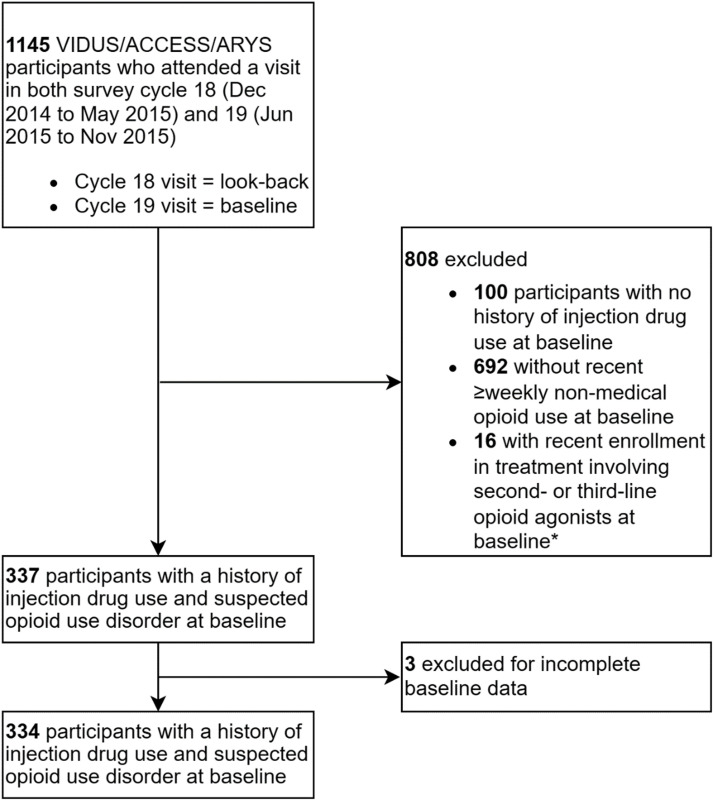
Flow of participants into study. *Notes:* VIDUS=*Vancouver Injection Drug Users Study*; ACCESS=*AIDS Care Cohort to Evaluate exposure to Survival Services* study; ARYS=*At-Risk Youth Study*; *Participants who reported current treatment with second- or third-line opioid agonist medications (e.g., slow-release oral morphine, injectable hydromorphone, or injectable diacetylmorphine) at their look-back visit were also excluded.

Fig. 2 shows that 334 VIDUS/ACCESS/ARYS participants were identified as eligible and included in the study cohort. Table 1 summarizes baseline participant characteristics, overall and by current first-line OAT status. At baseline, the median age of participants was 44 years (interquartile range [IQR]=32–52; range=18–70); 61% were cisgender men; 54% were white; and 50%, 30%, and 20% were VIDUS, ACCESS, and ARYS participants, respectively. Within the past six months, 98% of participants reported income from social assistance, 23% experienced homelessness, 16% experienced an overdose, 7% were incarcerated, and 97% injected drugs, of whom 98% injected opioids (64% daily, 34% weekly, and 2% less-than-weekly). Over half (55%) of participants reported recently injecting opioids and non-opioid drugs. One quarter of participants reported previously providing injection initiation assistance, with 25 (7%) recently providing such assistance. Prior (including current) first-line OAT was reported by 86% of participants.

**Table 1:** Baseline characteristics of 334 people who inject drugs with suspected opioid use disorder, overall and stratified by current first-line opioid agonist treatment (OAT) status.

		Current OAT at baseline	
Baseline characteristics	Overall (n = 334)	Yes (n = 177)	No (n = 157)
Age (in years), median (IQR)	44 (32–52)	44 (34–52)	43 (30–53)
Cisgender men, n (%)	203 (61)	99 (56)	104 (66)
Cohort, n (%)			
VIDUS	167 (50)	88 (50)	79 (50)
ACCESS	100 (30)	64 (36)	36 (23)
ARYS	67 (20)	25 (14)	42 (27)
Current opioid agonist treatment at prior visit, n (%)	175 (52)	146 (83)	29 (19)
History of providing injection initiation assistance, n (%)			
Yes, recent^a^	25 (7)	9 (5)	16 (10)
Yes, not recent^a^	60 (18)	25 (14)	35 (22)
No/never	249 (75)	143 (81)	106 (68)
Recent income sources^a^^,^^b^, n (%)			
Paid legal work^c^	81 (24)	37 (21)	44 (28)
Social assistance	326 (98)	174 (98)	152 (97)
Street-based activities^d^	100 (30)	45 (25)	55 (35)
Sex work	51 (15)	33 (19)	18 (12)
Illegal activities^e^	122 (37)	55 (31)	67 (43)
Recent homelessness^a^, n (%)	75 (23)	26 (15)	49 (31)
Recent incarceration^a^, n (%)	24 (7)	12 (7)	12 (8)
Recent non-fatal overdose^a^, n (%)	53 (16)	21 (12)	32 (20)
Recent frequency of opioid IDU^a^, n (%)			
Daily	205 (61)	86 (49)	119 (76)
Weekly	108 (32)	83 (47)	25 (16)
Less-than-weekly	5 (2)	5 (3)	0 (0)
None	16 (5)	3 (2)	13 (8)
Recent frequency of non-opioid IDU^a^^,^^f^, n (%)			
Daily	73 (22)	33 (19)	40 (26)
Weekly	74 (22)	40 (23)	34 (22)
Less-than-weekly	43 (13)	22 (12)	21 (13)
None	144 (43)	82 (46)	62 (40)
Recent public injection^a^, n (%)	171 (51)	79 (45)	92 (59)

*Notes:* IQR = interquartile range; IDU = injection drug use. All percentages are column percentages and sum may exceed 100% due to rounding.

a ‘Recent’ denotes behaviours, experiences, and/or activities over the past six months.

b Participants could select multiple income sources (of 5 categories presented).

c Regular job, temporary work, and/or self-employed.

d Panhandling, squeegeeing (window washing), or recycling.

e Drug dealing, theft, or other criminal activity.

f Includes cocaine (powder or rock/crack), crystal methamphetamine, benzodiazepines, and other non-opioid drugs.

At baseline, a lower proportion of participants on first-line OAT (versus those not on OAT) reported recent provision of injection initiation assistance, homelessness, overdose, daily injecting of opioids or non-opioids, and any public injecting over the past six months. Over 80% of participants on OAT at baseline reported current treatment at their look-back visit versus only 19% of participants not on OAT at baseline.

### 3.2 Follow-up and censoring

Overall, 50 (15%) participants were censored before their first semi-annual visit after baseline. The other 284 (85%) participants contributed 1114 follow-up visits (median [IQR]=5 [3–5] visits per participant) (Table 2). Of the 158 prematurely censored participants, 148 were censored for a missing outcome measurement (97% due to missed visit) and 10 were censored for initiating treatment with an alternative OAT medication.

**Table 2:** Frequency of recent injection initiation assistance provision, stratified by visit (*t*) and current first-line opioid agonist treatment status at prior visit (*t*-1), based on 334 people who inject drugs with suspected opioid use disorder from Vancouver, Canada—December 2014 to May 2018.

			Recent injection initiation assistance provision^b^ at visit *t*	
*t*	n^a^	Current OAT at visit *t*-1	Yes, n (%^c^)	No, n (%^c^)
0	334	Yes	11 (6.3)	164 (93.7)
		No	14 (8.8)	145 (91.2)
1	284	Yes	4 (2.6)	149 (97.4)
		No	7 (5.3)	124 (94.7)
2	245	Yes	5 (3.5)	139 (96.5)
		No	4 (4.0)	97 (96.0)
3	217	Yes	7 (5.4)	123 (94.6)
		No	8 (9.2)	79 (90.8)
4	192	Yes	6 (4.9)	117 (95.1)
		No	3 (4.4)	66 (95.7)
5	176	Yes	1 (0.9)	106 (99.1)
		No	5 (7.3)	64 (92.7)

OAT = Opioid agonist treatment (methadone or buprenorphine/naloxone).

a Number of remaining (uncensored) participants.

b Helped someone initiate injection drug use in the past six months.

c Row percentages (sum may exceed 100% due to rounding).

### 3.3 Opioid agonist treatment and injection initiation assistance provision

Table 2 summarizes the frequency of recent injection initiation assistance provision among participants by follow-up visit and current first-line OAT status (lagged to prior visit). The proportion of participants reporting current first-line OAT at their preceding visit ranged between 54 and 64% per follow-up visit. Of the 64 possible treatment trajectories between participants’ look-back visit and the final exposure assessment (*t*=4), the most frequently observed treatment history among uncensored participants was reporting current first-line OAT at every visit (41%; 79/192) followed by never reporting current OAT (21%; 41/192).

The proportion of participants reporting recent injection initiation assistance provision was low, irrespective of follow-up visit (range=3.4–6.9%). In total, there were 50 follow-up visits where recent injection initiation assistance provision was reported across 36 unique participants (median [IQR]=1 [1–2], range=1–4 follow-up visits with outcome per participant).

**Fig. 3:**
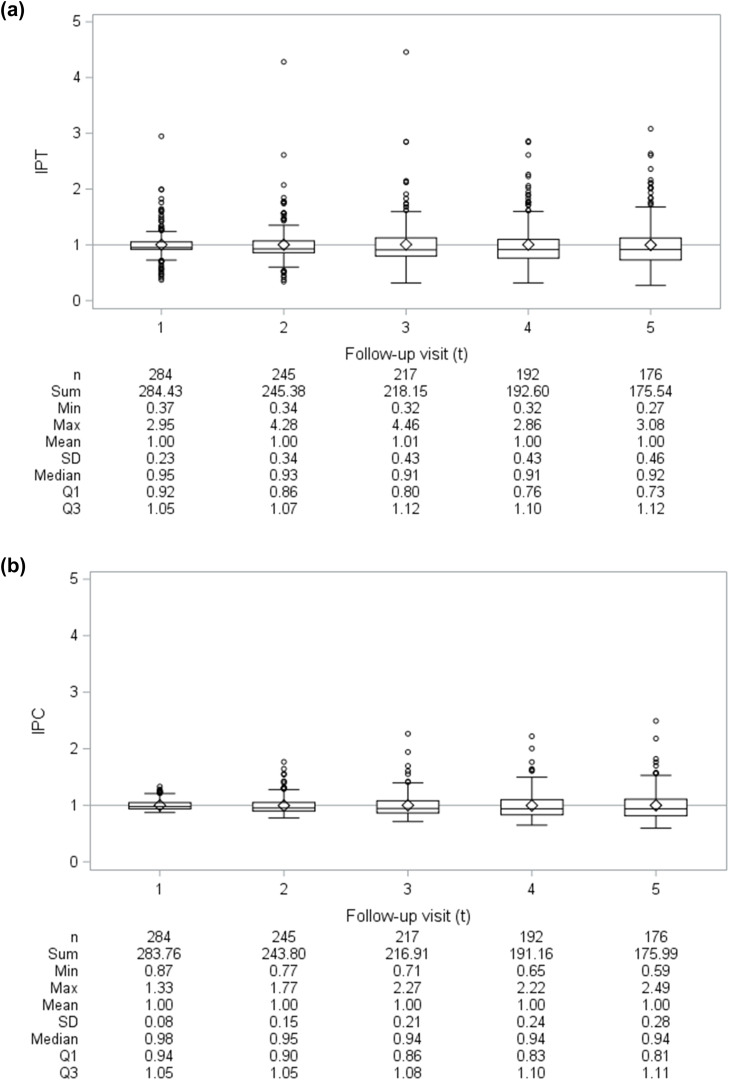

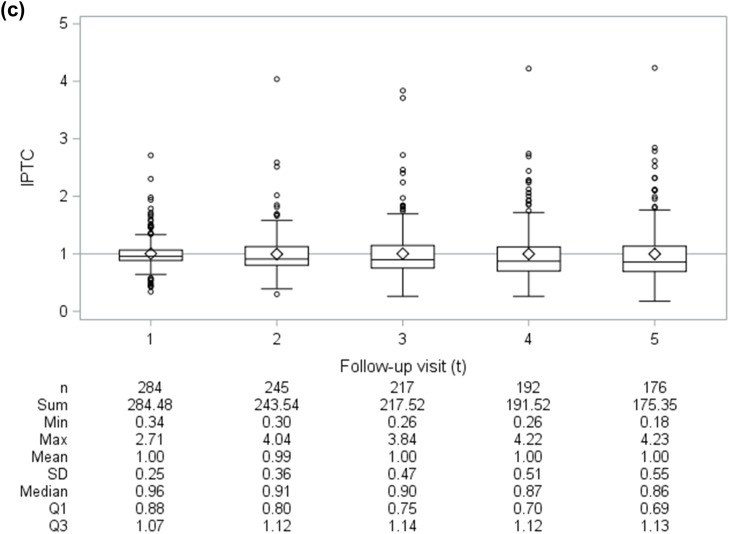
Distribution of (a) inverse-probability-of-treatment (IPT), (b) inverse-probability-of-censoring (IPC), and (c) inverse-probability-of-treatment-and-censoring (IPTC) weights over time. *Notes:* ◇ denotes mean value, while the horizontal reference line intersects with the Y-axis where the weight equals 1 (i.e., numerator equivalent to denominator); SD=standard deviation; Q1=first quartile; Q2=second quartile (median); Q3=third quartile.

Fig. 3 visualizes estimated weight distributions for the 1114 person-visit observations by 284 participants contributing to our outcome models, though all 334 eligible participants contributed to weight estimation. At each follow-up visit, the stabilized IPT, IPC, and IPTC weights appear well behaved, as mean values are (approximately) equal to 1 and no extreme weights were observed.

Table 3 presents our repeated measures log-binomial regression model results estimating the average effect of first-line OAT at visit *t*-1 on the risk of providing injection initiation assistance in the following six months (i.e., between visits (*t*-1, *t*]), conditional on treatment status at visit *t*-2. Based on the primary IPTC-weighted estimate, participants on first-line OAT at a given visit were, on average, 50% less likely than participants not on OAT to report helping someone initiate injecting in the following six months (RR=0.50, 95% CI=0.23–1.11). Weighting minimally impacted estimation, as the IPTC-weighted and unweighted estimates (RR=0.54, 95% CI=0.25–1.17) are of comparable magnitude and precision. Compared to the IPTC-weighted estimate, the IPT-weighted estimate was further from the null (RR=0.47, 95% CI=0.21–1.05) whereas the IPC-weighted estimate was closer to the null (RR=0.56, 95% CI=0.25–1.23). Non-parametric percentile bootstrapping generated 95% CI of similar width to the robust 95% CI (**Appendix Table 1**).

**Table 3:** Estimates of the average effect of current first-line opioid agonist treatment (OAT) at visit *t*-1 on risk of providing injection initiation assistance over the next six months (i.e., between visits (*t*-1, *t*]) based on 1114 person-visits (before weighting) by 284^a^ people who inject drugs with suspected opioid use disorder from Vancouver, Canada—December 2014 to May 2018.

		Effect of current OAT at visit *t*-1	
Model specifications^a^	n^b^ (# with outcome^b^)	Relative Risk	95% CI^c^
Unweighted	1114 (50)	0.54	0.25 to 1.17
IPC weighted	1112 (50)	0.56	0.25 to 1.23
IPT weighted	1116 (54)	0.47	0.21 to 1.05
IPTC weighted	1112 (53)	0.50	0.23 to 1.11

IPC = inverse-probability-of-censoring; IPT = inverse-probability-of-treatment; IPTC = inverse-probability-of-treatment-and-censoring; CI = confidence interval.

a All estimates obtained from log-binomial generalized estimating equations regression models with recent injection initiation assistance provision (outcome) regressed on fixed terms for follow-up visit (*t*=[1,2,3,4,5]), current OAT at visit *t*-1 (exposure), and current OAT at visit *t*-2 (covariate), with an unstructured working correlation structure for within-participant responses. All models based on 1114 person-visit observations (before weighting) by 284 unique participants. All other eligible participants (50/334) were censored by *t* = 1 (see Table 2) and only contributed to weight estimation.

b For weighted models, n and # with outcome rounded to nearest integer after application of weights.

c Based on robust variance estimator with clustering by subject.

Adding recent injection initiation assistance provision at visit *t*-1 to the IPTC weights yielded an equivalent short-term treatment effect estimate (IPTC-weighted n = 1111 person-visits [51 with outcome]; RR=0.49, 95% CI=0.22–1.13) to the primary IPTC-weighted analysis.

Table 4 summarizes our probabilistic bias analyses. The conventional unweighted estimate (RR=0.54) became slightly more protective following adjustment for exposure misclassification (median RR=0.48, 95% SI [total error]=0.20–1.12), exposure and covariate misclassification (median RR=0.51, 95% SI [total error]=0.22–1.14), and outcome misclassification (median RR=0.49, 95% SI [total error]=0.22–1.13). These results also suggest that random error had a larger contribution to interval width relative to uncertainty in the bias parameter distributions (systematic error).

**Table 4:** Results of probabilistic bias analyses adjusting for nondifferential misclassification of current opioid agonist treatment—measured at both visit *t*-1 (as exposure) and visit *t*-2 (as covariate)—and differential misclassification of recent injection initiation assistance provision (outcome).

Analysis	RR	95% Interval	Interval Width
Conventional (random error only)	0.54	0.25 to 1.17	4.68
Exposure misclassification			
Systematic error only	0.48	0.26 to 0.86	3.31
Total (systematic and random) error	0.48	0.20 to 1.12	5.60
Exposure and covariate misclassification^e^			
Systematic error only	0.51	0.29 to 0.89	3.07
Total (systematic and random) error	0.51	0.22 to 1.14	5.18
Outcome misclassification			
Systematic error only	0.49	0.31 to 0.80	2.58
Total (systematic and random) error	0.49	0.22 to 1.13	5.14

RR = Relative Risk.

^a^ All analyses unweighted, with outcome models specified as in primary regression analyses (see Table 3) with recent injection initiation assistance provision (outcome) regressed on terms for time (*t;* follow-up visit), current opioid agonist treatment at visit *t*-1 (exposure), and current opioid agonist treatment at visit *t*-2 (covariate).

^b^ Upper limit of interval (97.5th percentile value) divided by lower limit of interval (2.5th percentile value).

^c^ For conventional result, 95% Interval is 95% CI from Table 3.

^d^ For the bias-adjusted results, the reported RR is the median value across all converging iterations and the 95% Interval is Simulation Interval that incorporates systematic error (and random error where explicitly noted).

e Updated covariate value for a given person-visit observation where *t*=(1,2,3,4,5) based on lagged, bias-adjusted value of exposure for same participant at prior visit.

In a post hoc analysis, current first-line OAT (versus no OAT) was associated with a substantial reduction in the risk of providing injection initiation assistance within the next six months in participants with less-than-daily baseline opioid injecting (IPTC-weighted n = 468 person-visits [17 with outcome]; RR=0.15, 95% CI=0.05–0.44) but not in participants with daily baseline opioid injecting (IPTC-weighted n = 644 person-visits [36 with outcome]; RR=0.86, 95% CI=0.35–2.11).

## 4 Discussion

In this longitudinal cohort study, we evaluated whether first-line OAT (liquid methadone or sublingual buprenorphine/naloxone) in people who inject drugs with suspected opioid use disorder—characterized by frequent non-medical opioid use at baseline—reduces their short-term likelihood of helping others initiate injection drug use. According to our primary inverse-probability-weighted estimate, participants currently on first-line OAT were 50% less likely, on average, to help someone initiate injecting in the following six months versus participants not on medication for opioid use disorder; however, the corresponding 95% CI was imprecise, exhibiting compatibility with both a stronger beneficial effect (77% relative risk reduction) and weak, likely negligible detrimental effect (11% relative risk increase). Notably, the magnitude and precision of the estimated treatment effect were relatively consistent across unweighted and alternatively-weighted secondary analyses exploring violations of the no weight model misspecifications, no unmeasured confounding, and ignorable censoring assumptions underlying our inferences. Additionally, adjustments for self-reported outcome and treatment misclassification yielded slightly more protective effect estimates.

Our study is the first to use incidence data to demonstrate support for an effect of first-line OAT on subsequent injection initiation assistance provision in people who inject drugs with frequent non-medical opioid use. Despite intentional differences in design and analysis, the direction and magnitude of our primary effect estimate fits with prior cross-sectional associations involving the same Vancouver-based cohorts and outcome (recent injection initiation assistance provision) (Marks et al., 2021b; Mittal et al., 2019b). Using VIDUS/ACCESS/ARYS questionnaire data from 2014 to 2017, both Mittal et al. (1740 participants; 4.6% with outcome) and Marks et al. (1825 participants; 4.8% with outcome) found that people who inject drugs enrolled in first-line OAT within the past six months were less likely to report helping someone initiate injecting over the same period (odds ratio=0.52, 95% CI=0.31–0.87 and prevalence ratio=0.65, 95% CI=0.43–1.00, respectively), after adjusting for a similar set of potential confounders including age, gender, and recent injection drug use (Marks et al., 2021b; Mittal et al., 2019b). This relative consistency in point estimates suggests that the sources of potential bias we identified in previous studies—specifically, including treatment-ineligible participants and contemporaneously measured variables (Marks et al., 2021b; Mittal et al., 2019b)—and addressed in our primary analysis to improve validity may have minimally biased estimation. Collectively, this mounting evidence indicates support for increasing OAT coverage in people who inject drugs as a strategy to both reduce injection and associated harms among treated individuals and prevent incident injection events in the broader population of people who use drugs (Marks et al., 2021b, 2019; Meyers-Pantele et al., 2021; Mittal et al., 2019b).

While the direction and magnitude of our primary estimate offer support for a protective treatment effect, the imprecision of the corresponding 95% CI represents a key limitation that could be due to several factors. First and foremost, precision was clearly limited by the low number of outcomes contributing to the analysis as a result of our small sample size and suspected underreporting of injection initiation assistance provision because of stigma and poor recall (Guise et al., 2018; Mittal et al., 2019a). Precision could have been compromised further by self-reported treatment (exposure) misclassification, which can inflate standard errors and widen confidence intervals for treatment effect estimates (van Smeden et al., 2020).

Another explanation for the uncertainty in our primary result is treatment effect heterogeneity across subgroups constituting the overall study population (Corraini et al., 2017). Supporting this notion, our post hoc analysis results imply that current first-line OAT might substantially reduce the short-term risk of injection initiation assistance provision in participants with less-than-daily baseline opioid injecting but not in participants with daily baseline opioid injecting. While promising, the magnitude of the estimated treatment effect in the lower frequency opioid injecting subgroup is surely exaggerated due to an inadequate number of injection initiation assistance provision reports within OAT and covariate strata (sparse data bias) (Greenland et al., 2016). Conversely, the approximately null treatment effect in participants with daily opioid injecting could be owed to this subgroup being largely comprised of individuals with more severe opioid use disorder for whom methadone or buprenorphine/naloxone may not adequately reduce their opioid cravings, withdrawal symptoms, and non-medical opioid injecting (Fairbairn et al., 2019). Therefore, some participants in this subgroup may have continued injecting opioids frequently despite current or past first-line OAT (86% ever enrolled at baseline), thereby remaining at increased risk of providing injection initiation assistance versus the overall study population (Melo et al., 2018; Mittal et al., 2019b; Navarro et al., 2019; White et al., 2020). Such participants may have instead benefited from emerging alternative OAT medications (e.g., slow-release oral morphine or injectable hydromorphone) versus initiating, restarting, or continuing first-line OAT (Centre for Addiction and Mental Health (CAMH), 2021; Fairbairn et al., 2019). Although these alternative OAT medications were not a focus of this study as their use was heavily restricted during follow-up, they have become increasingly prescribed in Canada in recent years to expand treatment coverage amid an ongoing fentanyl-driven opioid crisis (Ivsins et al., 2020; Young et al., 2022). Future investigations might evaluate the effectiveness of alternative OAT medications on reducing non-medical opioid injecting and injection initiation assistance provision in people who inject opioids daily, including those with prior history of first-line OAT, given their elevated risk of exposing non-injectors to injection practices and helping them initiate injecting (Fairbairn et al., 2019).

There are other study limitations worth noting. The generalizability of our results to the intended target population (Vancouver-based people who inject drugs with opioid use disorder) is questionable, as the study cohort represents a convenience sample in which the prevalence of current first-line OAT (range=54–64%) far exceeds prior estimates of current OAT enrollment in North American people who inject drugs with opioid use disorder (range=11–34%) (Larney et al., 2017). Relatedly, we could not adjust for sources of bias affecting selection of participants into the study through weighting, which would require information on the sampling frame that is unavailable. Lastly, our study includes observations both before and during (2016-present) the fentanyl era of the opioid crisis in British Columbia. Knowledge of increasing adulteration of the unregulated drug supply with high-potency fentanyl and its analogues may have reinforced some participants’ attitudes against providing injection initiation assistance, resulting in reduced likelihood of engaging in this behavior over time independent of treatment status (Olding et al., 2019).

## Conclusions

Our findings suggest that first-line OAT may meaningfully reduce the short-term likelihood that people who inject drugs with frequent non-medical opioid use help others initiate injecting, particularly among individuals with less-than-daily opioid injecting. However, the extent of this presumed effect remains uncertain due to imprecise estimation and observed heterogeneity by baseline opioid injection frequency. Future investigations on this topic should prioritize recruiting a larger cohort, consider effect modification by baseline opioid injection frequency (and previous OAT history), and evaluate emerging alternative OAT medications, including in people who inject opioids daily given their increased risk of providing injection initiation assistance.

## Authors’ contributions

ZB, ACT, LR, and DW formulated the research question and hypothesis. ZB conducted the statistical analyses and wrote the initial draft. All authors contributed to the design, interpretation of the results, and manuscript revisions.

## Role of funding source

None.

## Ethics approval and consent to participate

This study was approved by the University of California San Diego Human Research Protection Program, the University of British Columbia/Providence Health Care and University of Toronto Research Ethics Boards (#27433).

## Consent for publication

Not applicable.

## Availability of data and materials

Data sharing and privacy agreements for VIDUS/ACCESS/ARYS prohibit the investigators from making the data set publicly available. Partial code has been provided in the Supplemental Materials.

## Declaration of Competing Interest

The authors declare that they have no conflicts of interest.

## Appendix Supplementary materials

Image, application 1

## References

[bib0001] Backmund M., Reimer J., Meyer K., Gerlach J.T., Zachoval R. (2005). Hepatitis C virus infection and injection drug users: prevention, risk factors, and treatment. Clin. Infect. Dis..

[bib0002] Ballinger G.A. (2004). Using generalized estimating equations for longitudinal data analysis. Organ Res, Methods.

[bib0003] Ben Hamida A., Rafful C., Jain S., Sun S., Gonzalez-Zuniga P., Rangel G., Strathdee S.A., Werb D. (2018). Non-injection drug use and injection initiation assistance among people who inject drugs in Tijuana, Mexico. J. Urban Health.

[bib0004] Bluthenthal R.N., Wenger L., Chu D., Lorvick J., Quinn B., Thing J.P., Kral A.H. (2015). Factors associated with being asked to initiate someone into injection drug use. Drug Alcohol Depend..

[bib0005] Bluthenthal R.N., Wenger L., Thing J., Chu D., Lorvick J., Quinn B., Kral A. (2015). Factors associated with precursors to initiating other people into injection drug use. Drug Alcohol Depend..

[bib0006] Bouck Z., Jain S., Sun X., Milloy M.-J., Werb D., Hayashi K. (2020). Recent incarceration and risk of first-time injection initiation assistance: a prospective cohort study of persons who inject drugs. Drug Alcohol Depend..

[bib0007] Bouck Z., Tricco A.C., Rosella L.C., Ling V., Gomes T., Tadrous M., Fox M.P., Scheim A.I., Werb D. (2022). Validation of self-reported opioid agonist treatment among people who inject drugs using prescription dispensation records. Epidemiology.

[bib0008] Bowles J.M., Jain S., Sun X., Strathdee S.A., DeBeck K., Milloy M.-J., Bouck Z., Werb D. (2021). Investigating a bidirectional relationship between overdose and provision of injection initiation assistance among persons who inject drugs in Vancouver, Canada and Tijuana, Mexico. Int. J. Drug Policy.

[bib0009] Brugal M.T., Barrio G., Fuente L.D.L., Regidor E., Royuela L., Suelves J.M. (2002). Factors associated with non-fatal heroin overdose: assessing the effect of frequency and route of heroin administration. Addiction.

[bib0010] Bruneau J., Ahamad K., Goyer M.-È., Poulin G., Selby P., Fischer B., Wild T.C., Wood E. (2018). Management of opioid use disorders: a national clinical practice guideline. CMAJ.

[bib0011] Bryant J., Treloar C. (2007). The gendered context of initiation to injecting drug use: evidence for women as active initiates. Drug Alcohol Rev..

[bib0012] Canadian Research Initiative in Substance Misuse (CRISM), 2018. National guideline for the clinical management of opioid use disorder.

[bib0013] Centre for Addiction and Mental Health (CAMH), 2021. Opioid agonist therapy: a synthesis of Canadian guidelines for treating opioid use disorder 52.

[bib0014] Cole S.R., Hernán M.A. (2008). Constructing inverse probability weights for marginal structural models. Am. J. Epidemiol..

[bib0015] Corraini P., Olsen M., Pedersen L., Dekkers O.M., Vandenbroucke J.P. (2017). Effect modification, interaction and mediation: an overview of theoretical insights for clinical investigators. Clin. Epidemiol..

[bib0016] Crofts N., Louie R., Rosenthal D., Jolley D. (1996). The first hit: circumstances surrounding initiation into injecting. Addiction.

[bib0017] Darke S. (2003). Heroin overdose: research and evidence-based intervention. J. Urban Health: Bull. New York Acad. Med..

[bib0018] Degenhardt L., Charlson F., Stanaway J., Larney S., Alexander L.T., Hickman M., Cowie B., Hall W.D., Strang J., Whiteford H., Vos T. (2016). Estimating the burden of disease attributable to injecting drug use as a risk factor for HIV, hepatitis C, and hepatitis B: findings from the Global Burden of Disease Study 2013. Lancet Infect. Dis..

[bib0019] Degenhardt L., Peacock A., Colledge S., Leung J., Grebely J., Vickerman P., Stone J., Cunningham E.B., Trickey A., Dumchev K., Lynskey M., Griffiths P., Mattick R.P., Hickman M., Larney S. (2017). Global prevalence of injecting drug use and sociodemographic characteristics and prevalence of HIV, HBV, and HCV in people who inject drugs: a multistage systematic review. The Lancet Glob. Health.

[bib0020] Des Jarlais D., Uuskula A., Talu A., Barnes D.M., Raag M., Arasteh K., Org G., Demarest D., Feelemyer J., Berg H., Tross S. (2019). Implementing an updated “Break the Cycle” intervention to reduce initiating persons into injecting drug use in an eastern European and a US “opioid epidemic” setting. AIDS Behav..

[bib0021] Des Jarlais D.C., Arasteh K., Barnes D.M., Feelemyer J., Berg H., Raag M., Talu A., Org G., Tross S., Uuskula A. (2021). A multistage process model of how a person who currently injects drugs comes to assist persons who do not inject with their first injections. Front. Sociol..

[bib0022] Dunlap B., Cifu A.S. (2016). Clinical management of opioid use disorder. JAMA.

[bib0023] Fairbairn N., Ross J., Trew M., Meador K., Turnbull J., MacDonald S., Oviedo-Joekes E., Le Foll B., Goyer M.-È., Perreault M., Sutherland C. (2019). Injectable opioid agonist treatment for opioid use disorder: a national clinical guideline. CMAJ.

[bib0024] Fox, M.P., MacLehose, R.F., Lash, T.L., 2022. Record level probabilistic bias analysis – SAS code [WWW Document]. Google Docs. URL https://drive.google.com/file/d/11fSicPHUfFy2x6evmTRf26awnmDuwF-1/view (accessed 3.25.22).

[bib0025] Fox M.P., MacLehose R.F., Lash T.L., Fox M.P., MacLehose R.F., Lash T.L. (2021). Applying Quantitative Bias Analysis to Epidemiologic Data.

[bib0026] Frajzyngier V., Neaigus A., Gyarmathy V.A., Miller M., Friedman S.R. (2007). Gender differences in injection risk behaviors at the first injection episode. Drug Alcohol Depend..

[bib0027] Garfein R.S., Doherty M.C., Monterroso E.R., Thomas D.L., Nelson K.E., Vlahov D. (1998). Prevalence and incidence of hepatitis C virus infection among young adult injection drug users. J. Acquir. Immune. Defic. Syndr. Hum. Retrovirol..

[bib0028] Garfein, R.S., Vlahov, D., Doherty, M.C., Nelson, K.E., 1996. Human immunodeficiency, and human T-lymphotropic viruses 86, 7. 10.2105/ajph.86.5.655 PMC1380472 8629715

[bib0029] Gicquelais R.E., Werb D., Marks C., Ziegler C., Mehta S.H., Genberg B.L., Scheim A.I. (2020). Prevalence and correlates of providing and receiving assistance with the transition to injection drug use. Epidemiol. Rev..

[bib0030] Goldsamt L.A., Harocopos A., Kobrak P., Jost J.J., Clatts M.C. (2010). Circumstances, pedagogy and rationales for injection initiation among new drug injectors. J. Community Health.

[bib0031] Gowing L., Farrell M.F., Bornemann R., Sullivan L.E., Ali R. (2011). Oral substitution treatment of injecting opioid users for prevention of HIV infection. Cochrane Database Syst. Rev..

[bib0032] Greenland S., Mansournia M.A., Altman D.G. (2016). Sparse data bias: a problem hiding in plain sight. BMJ i1981.

[bib0033] Guise A., Horyniak D., Melo J., McNeil R., Werb D. (2017). The experience of initiating injection drug use and its social context: a qualitative systematic review and thematic synthesis. Addiction.

[bib0034] Guise A., Melo J., Mittal M.L., Rafful C., Cuevas-Mota J., Davidson P., Garfein R.S., Werb D. (2018). A fragmented code: the moral and structural context for providing assistance with injection drug use initiation in San Diego, USA. Int. J. Drug Policy.

[bib0035] Harocopos A., Goldsamt L.A., Kobrak P., Jost J.J., Clatts M.C. (2009). New injectors and the social context of injection initiation. Int. J. Drug Policy.

[bib0036] Hernán M.A., Brumback B., Robins J.M. (2001). Marginal structural models to estimate the joint causal effect of nonrandomized treatments. J. Am. Stat. Assoc..

[bib0037] Hernán M.A., Brumback B.A., Robins J.M. (2002). Estimating the causal effect of zidovudine on CD4 count with a marginal structural model for repeated measures. Statist. Med..

[bib0038] Hernán M.A., Robins J.M. (2020).

[bib0039] Howe C.J., Cole S.R., Lau B., Napravnik S., Eron J.J. (2016). Selection bias due to loss to follow up in cohort studies. Epidemiology.

[bib0040] Ivsins A., Boyd J., Beletsky L., McNeil R. (2020). Tackling the overdose crisis: the role of safe supply. Int. J. Drug Policy.

[bib0041] Karki P., Shrestha R., Huedo-Medina T.B., Copenhaver M. (2016). The impact of methadone maintenance treatment on HIV risk behaviors among high-risk injection drug users: a systematic review. Evid. Based Med. Public Health.

[bib0042] Keogh R.H., Daniel R.M., VanderWeele T.J., Vansteelandt S. (2018). Analysis of longitudinal studies with repeated outcome measures: adjusting for time-dependent confounding using conventional methods. Am. J. Epidemiol..

[bib0043] Kerr T., Marsh D., Li K., Montaner J., Wood E. (2005). Factors associated with methadone maintenance therapy use among a cohort of polysubstance using injection drug users in Vancouver. Drug Alcohol Depend..

[bib0044] Khobzi N., Strike C., Cavalieri W., Bright R., Myers T., Calzavara L., Millson M. (2009). A qualitative study on the initiation into injection drug use: necessary and background processes. Addict. Res. Theory.

[bib0045] Kolla G., Strike C., Roy É., Altenberg J., Balian R., Silver R., Hunt N. (2015). Initiation stories: an examination of the narratives of people who assist with a first injection. Subst. Use Misuse.

[bib0046] Larney S., Peacock A., Leung J., Colledge S., Hickman M., Vickerman P., Grebely J., Dumchev K.V., Griffiths P., Hines L., Cunningham E.B., Mattick R.P., Lynskey M., Marsden J., Strang J., Degenhardt L. (2017). Global, regional, and country-level coverage of interventions to prevent and manage HIV and hepatitis C among people who inject drugs: a systematic review. Lancet Glob. Health.

[bib0047] Luongo N.M., Dong H., Kerr T.H., Milloy M.-J.S., Hayashi K., Richardson L.A. (2017). Income generation and attitudes toward addiction treatment among people who use illicit drugs in a Canadian setting. Addict. Behav..

[bib0048] Marks C., Borquez A., Jain S., Sun X., Strathdee S.A., Garfein R.S., Milloy M.-J., DeBeck K., Cepeda J.A., Werb D., Martin N.K. (2019). Opioid agonist treatment scale-up and the initiation of injection drug use: a dynamic modeling analysis. PLoS Med..

[bib0049] Marks C., Bouck Z., Jain S., Sun X., Strathdee S.A., Vickerman P., DeBeck K., Milloy M.-J., Hayashi K., Werb D. (2021). The impact of recent homelessness on the provision of injection drug use initiation assistance among persons who inject drugs in Tijuana, Mexico and Vancouver, Canada. Drug Alcohol Depend..

[bib0050] Marks C., Meyers S.A., Jain S., Sun X., Hayashi K., Gonzalez-Zuniga P., Strathdee S.A., Garfein R.S., Milloy M.J., DeBeck K., Cummins K., Werb D. (2021). Involvement of people who inject drugs in injection initiation events: a cross-sectional analysis identifying similarities and differences across three North American settings. BMJ Open.

[bib0051] Mattick R.P., Breen C., Kimber J., Davoli M. (2009). Methadone maintenance therapy versus no opioid replacement therapy for opioid dependence. Cochrane Database Syst. Rev..

[bib0052] Melo J.S., Garfein R.S., Hayashi K., Milloy M.J., DeBeck K., Sun S., Jain S., Strathdee S.A., Werb D. (2018). Do law enforcement interactions reduce the initiation of injection drug use? An investigation in three North American settings. Drug Alcohol Depend..

[bib0053] Meyers-Pantele S.A., Jain S., Sun X., Marks C., DeBeck K., Hayashi K., Strathdee S.A., Werb D. (2021). Gender and the first-time provision of injection initiation assistance among people who inject drugs across two distinct North American contexts: Tijuana, Mexico and Vancouver, Canada. Drug Alcohol Rev..

[bib0054] Meyers-Pantele, S.A., Mittal, M.L., Jain, S., Sun, S., Rammohan, I., Fairbairn, N., Milloy, M.-J., DeBeck, K., Hayashi, K., Werb, D., 2022. The influence of poly-drug use patterns on the association between opioid agonist treatment engagement and injecting initiation assistance 7. 10.1186/s13011-022-00470-6 PMC9118611 35590419

[bib0055] Mittal M.L., Guise A., Rafful C., Gonzalez-Zuniga P., Davidson Peter, Vashishtha D., Strathdee S.A., Werb D. (2019). Another person was going to do it”: the provision of injection drug use initiation assistance in a high-risk U.S.-Mexico border region. Subst. Use Misuse.

[bib0056] Mittal M.L., Jain S., Sun S., DeBeck K., Milloy M.J., Hayashi K., Hadland S.E., Werb D. (2019). Opioid agonist treatment and the process of injection drug use initiation. Drug Alcohol Depend..

[bib0057] Mittal M.L., Vashishtha D., Sun S., Jain S., Cuevas-Mota J., Garfein R., Strathdee S.A., Werb D. (2017). History of medication-assisted treatment and its association with initiating others into injection drug use in San Diego, CA. Subst. Abuse Treat Prev. Policy.

[bib0058] Moodie E.E.M., Delaney J.A.C., Lefebvre G., Platt R.W. (2008). Missing confounding data in marginal structural models: a comparison of inverse probability weighting and multiple imputation. Int. J. Biostat..

[bib0059] Navarro S., Kral A.H., Strike C.S., Simpson K., Wenger L., Bluthenthal R.N. (2019). Factors associated with frequency of recent initiation of others into injection drug use among people who inject drugs in Los Angeles and San Francisco, CA, USA, 2016-17. Subst. Use Misuse.

[bib0060] Noroozi M., Higgs P., Bayani A., Armoon B., Astaneh A.N., Moghaddam L.F., Askari M. (2020). Non-fatal overdose among people who inject drugs in Tehran, Iran. Subst. Abuse Treat. Prev. Policy.

[bib0061] Olding M., Werb D., Guise A., Small W., McNeil R. (2019). Navigating social norms of injection initiation assistance during an overdose crisis: a qualitative study of the perspectives of people who inject drugs (PWID) in Vancouver, Canada. Int. J. Drug Policy.

[bib0062] Oviedo-Joekes E., Guh D., Brissette S., Marchand K., MacDonald S., Lock K., Harrison S., Janmohamed A., Anis A.H., Krausz M., Marsh D.C., Schechter M.T. (2016). Hydromorphone compared with diacetylmorphine for long-term opioid dependence: a randomized clinical trial. JAMA Psychiatry.

[bib0063] Rhodes T., Bivol S., Scutelniciuc O., Hunt N., Bernays S., Busza J. (2011). Narrating the social relations of initiating injecting drug use: transitions in self and society. Int. J. Drug Policy.

[bib0064] Richardson D.B., Kinlaw A.C., MacLehose R.F., Cole S.R. (2015). Standardized binomial models for risk or prevalence ratios and differences. Int. J. Epidemiol..

[bib0065] Robins J.M., Hernán M.A., Brumback B. (2000). Marginal structural models and causal inference in epidemiology. Epidemiology.

[bib0066] Rotondi N.K., Strike C., Kolla G., Rotondi M.A., Rudzinski K., Guimond T., Roy É. (2014). Transition to injection drug use: the role of initiators. AIDS Behav..

[bib0067] Schuster T., Lowe W.K., Platt R.W. (2016). Propensity score model overfitting led to inflated variance of estimated odds ratios. J. Clin. Epidemiol..

[bib0068] Simpson K.A., Kral A.H., Goldshear J.L., Wenger L., Strike C.S., Bluthenthal R.N. (2020). Reasons for assisting with injection initiation: results from a large survey of people who inject drugs in Los Angeles and San Francisco, California. Drug Alcohol Depend..

[bib0069] Small W., Fast D., Krusi A., Wood E., Kerr T. (2009). Social influences upon injection initiation among street-involved youth in Vancouver, Canada: a qualitative study. Subst. Abuse Treat. Prev. Policy.

[bib0070] Socías M.E., Ti L., Wood E., Nosova E., Hull M., Hayashi K., Debeck K., Milloy M.-J. (2019). Disparities in uptake of direct-acting antiviral therapy for hepatitis C among people who inject drugs in a Canadian setting. Liver Int..

[bib0071] Socías M.E., Wood E., Kerr T., Nolan S., Hayashi K., Nosova E., Montaner J., Milloy M.-J. (2018). Trends in engagement in the cascade of care for opioid use disorder, Vancouver, Canada, 2006–2016. Drug Alcohol Depend..

[bib0072] Socías M.E., Wood E., McNeil R., Kerr T., Dong H., Shoveller J., Montaner J., Milloy M.-J. (2017). Unintended impacts of regulatory changes to British Columbia Methadone Maintenance Program on addiction and HIV-related outcomes: an interrupted time series analysis. Int. J. Drug Policy.

[bib0073] Strike C., Rotondi M., Kolla G., Roy É., Rotondi N.K., Rudzinski K., Balian R., Guimond T., Penn R., Silver R.B., Millson M., Sirois K., Altenberg J., Hunt N. (2014). Interrupting the social processes linked with initiation of injection drug use: results from a pilot study. Drug Alcohol Depend..

[bib0074] Tagliaro F., Battisti Z.D., Smith F.P., Marigo M. (1998). Death from heroin overdose: findings from hair analysis. Lancet North Am. Ed..

[bib0075] Uusküla A., Barnes D.M., Raag M., Talu A., Tross S., Des Jarlais D.C. (2018). Frequency and factors associated with providing injection initiation assistance in Tallinn. Estonia. Drug Alcohol Depend..

[bib0076] van Haastrecht H.J.A., van Ameijden E.J.C., van den Hoek J.A.R., Mientjes G.H.C., Bax J.S., Coutinho R.A. (1996). Predictors of mortality in the Amsterdam cohort of human immunodeficiency virus (HIV)-positive and HIV-negative drug users. Am. J. Epidemiol..

[bib0077] van Smeden M., Lash T.L., Groenwold R.H.H. (2020). Reflection on modern methods: five myths about measurement error in epidemiological research. Int. J. Epidemiol..

[bib0078] VanderWeele T.J., Hawkley L.C., Thisted R.A., Cacioppo J.T. (2011). A marginal structural model analysis for loneliness: implications for intervention trials and clinical practice. J. Consult. Clin. Psychol..

[bib0079] Vashishtha D., Mittal M.L., Werb D. (2017). The North American opioid epidemic: current challenges and a call for treatment as prevention. Harm. Reduct. J..

[bib0080] Volkow N.D., Frieden T.R., Hyde P.S., Cha S.S. (2014). Medication-assisted therapies — Tackling the opioid-overdose epidemic. N. Engl. J. Med..

[bib0081] Werb D., Bluthenthal R.N., Kolla G., Strike C., Kral A.H., Uusküla A., Des Jarlais D. (2018). Preventing injection drug use initiation: state of the evidence and opportunities for the future. J. Urban Health.

[bib0082] Werb D., Garfein R., Kerr T., Davidson P., Roux P., Jauffret-Roustide M., Auriacombe M., Small W., Strathdee S.A. (2016). A socio-structural approach to preventing injection drug use initiation: rationale for the PRIMER study. Harm. Reduct. J..

[bib0083] White R.H., O'Rourke A., Bluthenthal R.N., Kral A.H., Kilkenny M.E., Hazelett T.D., Sherman S.G., Allen S.T. (2020). Initiating persons into injection drug use in rural West Virginia, USA. Subst. Use Misuse.

[bib0084] Witteveen E., Van Ameijden E.J.C., Schippers G.M. (2006). Motives for and against injecting drug use among young adults in Amsterdam: qualitative findings and considerations for disease prevention. Subst. Use Misuse.

[bib0085] Young S., Kolla G., McCormack D., Campbell T., Leece P., Strike C., Srivastava A., Antoniou T., Bayoumi A.M., Gomes T. (2022). Characterizing safer supply prescribing of immediate release hydromorphone for individuals with opioid use disorder across Ontario, Canada. Int. J. Drug Policy.

