# Supplementary material for: First-line opioid agonist treatment as prevention against assisting others in initiating injection drug use: A longitudinal cohort study of people who inject drugs in Vancouver, Canada
Source: Drug Alcohol Depend Rep. 2023 May 25;7:100168. doi: 10.1016/j.dadr.2023.100168 (PMC10311194; doi:10.1016/j.dadr.2023.100168)
Supplement: Supplementary file 1 [file mmc1.pdf]

SUPPLEMENTAL MATERIALS

Appendix 1. Alternative opioid agonist medications for opioid use disorder. p2-3.

Appendix 2. Estimation of the treatment selection and censoring weights. p4-10.

Appendix 3. Details of the external validation data used in the treatment (exposure) misclassification bias analysis. p11.

Appendix 4. SAS code for treatment misclassification—exposure, A_{it-1} and covariate, A_{it-2}—probabilistic bias analyses. p12-17.

Appendix Figure 1. Distributions for sensitivity and specificity of exposure (current first-line opioid agonist treatment at visit *t*-1) based on 10,000 iterations. p18.

Appendix 5. SAS code for outcome misclassification probabilistic bias analysis. p19-22.

Appendix Figure 2. Distributions for sensitivity and specificity of outcome (recent injection initiation assistance provision at visit *t*) by reported exposure status (current first-line opioid agonist treatment [OAT] at visit *t*-1) based on 10,000 iterations. p23.

Appendix Table 1. Alternative 95% CI calculated using non-parametric percentile bootstrapping with individual participants as cluster sampling units. p24.

Appendix 1. Alternative opioid agonist medications for opioid use disorder.

This section describes how we identified participants on alternative opioid agonist medications for opioid use disorder at baseline (for exclusion) and during follow-up (for censoring).

Baseline

All study participants completed their baseline visit between June 2015 and November 2015, a six-month period that directly corresponds with VIDUS/ACCESS/ARYS survey cycle 19. Participants were asked about their history of treatment for substance use disorders, both ever and recently (i.e., within the past six months). At baseline, there were a limited number of scenarios in which a participant could have received recent treatment for opioid use disorder involving slow-release oral morphine, oral hydromorphone, or an injectable opioid agonist (hydromorphone or diacetylmorphine)—all opioid agonist medications. First, participants in VIDUS/ACCESS/ARYS who also participated in the *Study to Assess Longer-term Opioid Medication Effectiveness* (SALOME) clinical trial could have reported recent treatment with one of these medications as of their baseline visit.[1] In brief, the SALOME trial recruited 202 participants from the Greater Vancouver Area, all with chronic heroin addiction unresponsive to available opioid agonist treatments (i.e., methadone and buprenorphine/naloxone) and randomized them 1:1 to receive either injectable diacetylmorphine (prescription heroin; n = 102) or injectable hydromorphone (n = 100).[1] The treatment phase of the trial concluded in early 2015.[1] Furthermore, some SALOME participants were approved by Health Canada to continue treatment involving injectable opioids (diacetylmorphine or hydromorphone) or oral hydromorphone beyond the trial’s conclusion.[2] Second, as of November 2014, slow-release oral morphine (once-daily formulation; brand name Kadian) was approved by Indigenous Services Canada’s Non-Insured Health Benefits (NIHB) Program for treatment of opioid use disorder in patients for whom methadone or buprenorphine/naloxone are ineffective or contra-indicated.[3] In total, we identified and excluded 12 participants who were recently exposed to alternative medications for opioid use disorder at baseline (exclusion criterion), all of whom indicated “other” past six-month treatment for alcohol or drug use with specification of “SALOME” as their other treatment when prompted. None of these 12 participants explicitly indicated what treatment they received as part of SALOME; therefore, we conservatively assumed that these participants recently received treatment involving oral hydromorphone or an injectable opioid agonist due to their ongoing or prior participation in SALOME.[1] Notably, no participants reported recent (or current) treatment with slow-release oral morphine at baseline.

Follow-up

Over follow-up, we applied a similar strategy to identify and censor participants who recently initiated one of these alternative opioid agonist medications for opioid use disorder. Participants who indicated "SALOME" when asked to specify their "other" recent drug treatment at a given follow-up visit were censored (assumed to have continued access to treatment with oral hydromorphone or an injectable opioid agonist through prior SALOME participation). Additionally, participants who explicitly reported recent treatment with slow-release oral morphine, hydromorphone (oral or injectable), or injectable diacetylmorphine at a given follow-up visit were censored. Specific response options to indicate recent enrollment in treatment involving any of these medications were not added until follow-up survey cycle 22 (December 2016 and May 2017) towards the end of our study period. During follow-up, British Columbia's publicly-funded provincial drug plan (B.C. PharmaCare) first began to provide coverage for slow-release oral morphine as treatment for opioid use disorder as of June 5th, 2017 (i.e., within the second last VIDUS/ACCESS/ARYS survey cycle [23] included in the study period and following the second last treatment assessment in cycle 22), which provides some insight into why this medication was infrequently initiated among participants during follow-up.[3]

Appendix 2. Estimation of the treatment selection and censoring weights.

Notation

Consistent with the main text, let *i* index individual participants (*i*=[1 to 334]); *t* or *k* (used interchangeably) denotes visit (*t*=[-1,0,1,2,3,4,5]; where *t*=0 is baseline); A_{it-1} is the focal exposure, indicating whether a participant reported current opioid agonist treatment at visit *t*-1 (1=yes/0=no); an overbar denotes the observed history of a variable, e.g., $\bar{\mathrm{A}}_{it-2}$ represents a participant's observed treatment history from their look-back visit through visit *t*-2, i.e., $\bar{\mathrm{A}}_{it-2}$=($\mathrm{A}_{i(-1)}, \mathrm{A}_{i0}, \ldots, \mathrm{A}_{it-2}$); C_{it} indicates whether a participant was censored by visit *t* (1=yes/0=no) for any reason (i.e., missed outcome measurement or starting an alternative opioid agonist medication for opioid use disorder); V_{i0} is the set of measured baseline-fixed covariates (age, gender, and cohort); and L_{it-1} is the set of time-varying covariates measured at visit *t*-1 (recent homelessness, recent incarceration, recent income from paid legal work, recent income from street-based activities, recent income from sex work, recent income from illegal activities, recent non-fatal overdose, recent frequency of opioid injection drug use, recent frequency of non-opioid injection drug use, and recent public injection). We note that the indicator variable capturing any recent income from social assistance was purposefully excluded from the time-varying covariate set due to a lack of variation in participants' responses at each visit (e.g., 98% of participants reported income from social assistance at baseline).

Estimation of treatment weights

The inverse-probability-of-treatment (IPT) weights or 'treatment' weights, $\mathrm{SW}_{it}^{\mathrm{A}}$, were calculated using the following formula:

$$\mathrm{SW}_{it}^{\mathrm{A}} = \prod_{k=1}^{t} \frac{\Pr[\mathrm{A}_{ik-1} | \bar{\mathrm{A}}_{ik-2,} \bar{\mathrm{C}}_{ik-1} = \bar{0}]}{\Pr[\mathrm{A}_{ik-1} | \bar{\mathrm{A}}_{ik-2}, \bar{\mathrm{C}}_{ik-1} = \bar{0}, \mathrm{V}_{i0}, \bar{\mathrm{L}}_{ik-1}]},$$

Where *t*=(1,2,3,4,5). To estimate the numerator and denominator components of the treatment weights, we independently fit two pooled logistic regression models.[4,5] The fitted denominator model was specified as:

$$\mathrm{logit}(\Pr[\mathrm{A}_{ik-1} = 1 | \bar{\mathrm{A}}_{ik-2}, \bar{\mathrm{C}}_{ik-1} = \bar{0}, \mathrm{V}_{i0}, \bar{\mathrm{L}}_{ik-1}]) = \beta_{0k} + \beta_1 \mathrm{A}_{ik-2} + \beta_2 \mathrm{V}_{i0} + \beta_3 \mathrm{L}_{ik-1},$$

where *k*=(1,2,3,4,5). The right-hand side of the pooled logistic regression was specified by applying the backdoor path criterion based on the implied relationships between measured variables in **Figure 1**.

The corresponding numerator model was of the form:

$$\mathrm{logit}(\Pr[\mathrm{A}_{ik-1} = 1 | \bar{\mathrm{A}}_{ik-2}, \bar{\mathrm{C}}_{ik-1} = \bar{0}]) = \beta_{0k} + \beta_1 \mathrm{A}_{ik-2},$$

where *k*=(1,2,3,4,5).

Estimation of censoring weights

The inverse-probability-of-censoring (IPC) or 'censoring' weights, SW_{it}^{C}, were calculated using the following formula:

$$SW_{it}^{C} = \prod_{k=1}^{t} \frac{\Pr[C_{ik} = 0 | \bar{C}_{ik-1} = \bar{0}, \bar{A}_{ik-1}]}{\Pr[C_{ik} = 0 | \bar{C}_{ik-1} = \bar{0}, \bar{A}_{ik-1}, V_{i0}, \bar{L}_{ik-1}]},$$

Where *t*=(1,2,3,4,5). Our approach for estimating the censoring weights mirrored our estimation of the treatment selection weights, with censoring treated as another time-varying "exposure".[4,6,7] We separately estimated the numerator and denominator components of the weight using pooled logistic regression. In both models, censoring was treated as a failure-time variable with participants no longer contributing person-visit observations beyond the visit at which they were first censored.[4] The fitted denominator pooled logistic regression model, where *k*=(1,2,3,4,5), was:

$$\text{logit}(\Pr[C_{ik} = 0 | \bar{C}_{ik-1} = \bar{0}, \bar{A}_{ik-1}, V_{i0}, \bar{L}_{ik-1}]) = \beta_{0k} + \beta_1 A_{ik-1} + \beta_2 V_{i0} + \beta_3 L_{ik-1},$$

and the fitted numerator pooled logistic regression model, where *k*=(1,2,3,4,5), was:

$$\text{logit}(\Pr[C_{ik} = 0 | \bar{C}_{ik-1} = \bar{0}, \bar{A}_{ik-1}]) = \beta_{0k} + \beta_1 A_{ik-1}$$

SAS code

The following SAS code was used to estimate the treatment and censoring weights:

```
/*NOTE ON VARIABLES USED:
Exposure/treatment at visit t (OAT_curr)
Outcome at visit t (pIIA_p6m)
Measured time-independent baseline covariates at visit t include:
      age (in years, treated as continuous; varname = age_bl
      self-identified gender (cisgender man: yes or no; varname = gender_bl)
      and cohort (VIDUS, ACCESS, or ARYS; varname = cohort)
Measured time-varying covariates at visit t include:
      recent homelessness (varname = homeless_l6m),
      recent incarceration (varname = jail_l6m),
      recent income from paid legal work (varname = employment_l6m),
      recent income from street-based activities (varname = street_inc_l6m),
      recent income from sex work (varname = sexwork_inc_l6m),
      recent income from illegal activities (varname = ill_inc_l6m),
      recent non-fatal overdose (varname = overdose_l6m),
      recent frequency of opioid injection drug use (varname = injop_l6m)
      recent frequency of non-opioid injection drug use (varname =
      injnop_l6m),
      and
      recent public injection (injpub_l6m).

      Values of these same variables at visit t-1 have the same varname with
added lag_: prefix. */
```

```
*ESTIMATE TREATMENT WEIGHTS;

/*(A) for A(t) where t in 0,1,2,3,4*/
      *Denominator model;
proc logistic data=PhD_aim2.bl19_AAV_long_lag;
where t in (0,1,2,3,4) and t<=visits; /*analytic n=1272=participants not
censored as of visit t (i.e., have complete baseline data)*/
class t(ref="0") cohort(ref="VIDUS2") gender_bl(ref="0")
homeless_l6m(ref="No") jail_l6m(ref="No")
      injop_l6m(ref="None") injnop_l6m(ref="None") overdose_l6m(ref="No")
      employment_l6m(ref="0") street_inc_l6m(ref="0") ill_inc_l6m(ref="0")
      sexwork_inc_l6m(ref="0")
      injpub_l6m(ref="0") lag_OAT_curr(ref="0")
      /param=ref;
model OAT_curr(event="Yes") /*At*/ = t /*time-specific intercepts*/ age
gender_bl cohort /*V0*/
      homeless_l6m jail_l6m overdose_l6m injop_l6m injnop_l6m injpub_l6m
      employment_l6m street_inc_l6m ill_inc_l6m sexwork_inc_l6m /*Lt*/
      lag_OAT_curr /*At-1*/;
output out=IPT_denom p=dprob;
run;

      *Numerator model;
proc logistic data=IPT_denom;
where t in (0,1,2,3,4) and dprob ne .; /*to ensure same analytic n as denom
(1272 obs)*/
class t(ref="0") lag_OAT_curr(ref="0")/param=ref;
model OAT_curr(event="Yes") /*At*/ = t /*time-specific intercepts*/
lag_OAT_curr /*At-1*/;
output out=IPT_numanddenom p=nprob;
run;

data IPT_numanddenom_2;
set IPT_numanddenom;
if OAT_curr=1 then do;
d_At=dprob;
n_At=nprob;
end;
if OAT_curr=0 then do;
d_At=1-dprob;
n_At=1-nprob;
end;
run;

/*calculate IPT per person-visit*/
proc sort data=IPT_numanddenom_2; by id t; run;
data PhD_aim2.IPT_f;
set IPT_numanddenom_2;
by ID t;
retain num_At den_At;
if first.ID then do; num_At=1; den_At=1; end;
num_At=num_At*n_At;
den_At=den_At*d_At;
IPT=num_At/den_At;
run;
proc means data=PhD_aim2.IPT_f n sum min max mean std;
var IPT;
run;
```

```
*ESTIMATE CENSORING WEIGHTS;

data c_pooled;
set PhD_aim2.bl19_AAV_long_lag;
if t eq 4 then delete;
if t<visits then C=0;
if t=visits then C=1; /*censored as of t+1 (that is, no visits beyond visit
t)*/
if t>visits then delete; /*removes previously censored participants from
risk set*/
run; *1272 obs;

      *Denominator model;
proc logistic data=c_pooled;
where t in (0,1,2,3,4); /*modelling c=C(t+1) as response variable (to
include those with C(t+1)=1 or 0), # obs=1272 (same as treatment
modelling)*/
class c t(ref="0") gender_bl(ref="0") cohort(ref="VIDUS2")
OAT_curr(ref="No") homeless_l6m(ref="No") jail_l6m(ref="No")
      injop_l6m(ref="None”) injnop_l6m(ref="None") overdose_l6m(ref="No")
      employment_l6m(ref="0") street_inc_l6m(ref="0") ill_inc_l6m(ref="0")
      sexwork_inc_l6m(ref="0")injpub_l6m(ref="0")/param=ref;
model c(event="0") /*C(t+1)=0*/ = t age gender_bl cohort /*V0*/ OAT_curr
/*At*/ homeless_l6m jail_l6m overdose_l6m injop_l6m injnop_l6m injpub_l6m
      employment_l6m street_inc_l6m ill_inc_l6m sexwork_inc_l6m /*Lt*/;
output out=IPC_denom (keep=ID c t d_drop OAT_curr) p=d_drop;
run;

      *Numerator model = pooled logistic regression;
proc logistic data=IPC_denom;
where t in (0,1,2,3,4) and d_drop ne .; /*to ensure same analytic n as
denom*/
class c t(ref="0") OAT_curr(ref="No")/param=ref;
model c(event="0") = t OAT_curr /*At*/;
output out=IPC_numanddenom (keep=ID c t n_drop d_drop) p=n_drop;
run;

/*Calculate cumulative probabilities for stabilized weights (IPC) at each
visit*/
proc sort data=IPC_numanddenom; by ID t; run;
data IPC_numanddenom2;
set IPC_numanddenom;
by ID t;
retain num_drop den_drop;
if first.ID then do; num_drop=1; den_drop=1; end;
num_drop=num_drop*n_drop;
den_drop=den_drop*d_drop;
if c=0 then IPC=num_drop/den_drop;
if c=1 then IPC=.;
run;
proc means data=IPC_numanddenom2 n nmiss sum min mean max std p1 p25 p50 p75
p99;
where c=0;
var IPC;
run;
```

```
*next, merge above with IPT_f dataset (first will have to recode t in (0-4)
to t+1 so that t in (1-5) for IPC_f dataset;
data IPC_f;
set IPC_numanddenom2(where=(IPC ne .));
t2=t+1; /*shift up*/
keep t2 id IPC;
run;
data IPC_f_2;
set IPC_f;
t=t2;
drop t2;
run;
data IPT_f;
set PhD_aim2.IPT_f;
t2=t+1;
keep t2 id IPT num_At den_At;
run;
data IPT_f_2;
set IPT_f;
t=t2;
drop t2;
run;
proc sort data=IPC_f_2;
by id t;
proc sort data=IPT_f_2;
by id t;
data IPTC_1;
merge IPT_f_2(in=ina) IPC_f_2(in=inb);
by id t;
if ina and inb then output IPTC_1;
run;

*last, calculate final stabilized IPTC weights per follow-up observation;
data PhD_aim2.IPTC_f;
set IPTC_1;
IPTC=IPT*IPC;
run;
proc means data=PhD_aim2.IPTC_f n sum mean stddev median min q1 q3 p1 p99
max;
var IPTC;
run;
proc means data=PhD_aim2.IPTC_f n sum mean stddev median min q1 q3 p1 p99
max;
class t;
var IPTC;
run;
```

Fitted weight denominator models

Below are the resulting SAS Analysis of Maximum Likelihood Estimates outputs for the fitted treatment and censoring weight denominator logistic regression models, indicating the degree of association on the log odds scale between included covariates with treatment assignment at visit *t*-1 and censoring by visit *t*, respectively. ‘Estimate’ values are the estimated model coefficient per included covariate.

(a) Fitted treatment weight denominator model

Analysis of Maximum Likelihood Estimates

Parameter		DF	Estimate	Standard Error	Wald Chi-Square	Pr > ChiSq
**Intercept**		1	-0.7492	0.5741	1.7030	0.1919
**t**	**1**	1	0.1917	0.2407	0.6346	0.4257
**t**	**2**	1	0.0213	0.2498	0.0073	0.9319
**t**	**3**	1	0.1694	0.2609	0.4214	0.5163
**t**	**4**	1	-0.2632	0.2674	0.9686	0.3250
**age**	**+1 year**	1	-0.00631	0.0103	0.3774	0.5390
**gender_bl**	**Cis man**	1	-0.2779	0.1924	2.0859	0.1487
**cohort**	**ACCESS**	1	0.00755	0.1903	0.0016	0.9683
**cohort**	**ARYS**	1	-0.2903	0.3123	0.8641	0.3526
**homeless_l6m**	**Yes**	1	-0.1055	0.2390	0.1947	0.6591
**jail_l6m**	**Yes**	1	0.4080	0.3426	1.4179	0.2338
**overdose_l6m**	**Yes**	1	-0.3297	0.2186	2.2745	0.1315
**injop_l6m**	**<weekly**	1	0.8177	0.4015	4.1479	0.0417
**injop_l6m**	**>=weekly**	1	0.6539	0.3009	4.7226	0.0298
**injop_l6m**	**Daily**	1	-0.5738	0.2681	4.5821	0.0323
**injnop_l6m**	**<weekly**	1	0.1919	0.2684	0.5114	0.4746
**injnop_l6m**	**>=weekly**	1	0.0213	0.2238	0.0090	0.9242
**injnop_l6m**	**Daily**	1	-0.1328	0.2312	0.3298	0.5658
**injpub_l6m**	**Yes**	1	-0.2129	0.1996	1.1371	0.2863
**employment_l6m**	**Yes**	1	-0.1337	0.1988	0.4522	0.5013
**street_inc_l6m**	**Yes**	1	0.1012	0.1877	0.2907	0.5898
**ill_inc_l6m**	**Yes**	1	-0.0293	0.1931	0.0230	0.8796
**sexwork_inc_l6m**	**Yes**	1	0.0772	0.2637	0.0857	0.7697
**lag_OAT_curr**	**Yes**	1	3.3312	0.1675	395.6508	<.0001

(b) Fitted censoring weight denominator model

Analysis of Maximum Likelihood Estimates

Parameter		DF	Estimate	Standard Error	Wald Chi-Square	Pr > ChiSq
**Intercept**		1	1.1159	0.6255	3.1824	0.0744
**t**	**1**	1	0.0307	0.2418	0.0161	0.8990
**t**	**2**	1	0.2186	0.2635	0.6884	0.4067
**t**	**3**	1	0.1604	0.2764	0.3367	0.5617
**t**	**4**	1	0.5942	0.3136	3.5904	0.0581
**age**	**+1 year**	1	0.0128	0.0111	1.3356	0.2478
**gender_bl**	**Cis man**	1	-0.1870	0.2046	0.8350	0.3608
**cohort**	**ACCESS**	1	-0.1108	0.2087	0.2818	0.5955
**cohort**	**ARYS**	1	-0.2008	0.3212	0.3908	0.5319
**OAT_curr**	**Yes**	1	0.4128	0.1890	4.7690	0.0290
**homeless_l6m**	**Yes**	1	0.1787	0.2426	0.5428	0.4613
**jail_l6m**	**Yes**	1	0.0897	0.3537	0.0643	0.7998
**overdose_l6m**	**Yes**	1	0.00321	0.2288	0.0002	0.9888
**injop_l6m**	**<weekly**	1	-0.1993	0.4016	0.2461	0.6198
**injop_l6m**	**>=weekly**	1	0.00974	0.3309	0.0009	0.9765
**injop_l6m**	**Daily**	1	0.1157	0.3008	0.1479	0.7006
**injnop_l6m**	**<weekly**	1	0.7365	0.3239	5.1709	0.0230
**injnop_l6m**	**>=weekly**	1	0.7716	0.2709	8.1107	0.0044
**injnop_l6m**	**Daily**	1	-0.0875	0.2260	0.1498	0.6987
**injpub_l6m**	**Yes**	1	-0.5188	0.2157	5.7826	0.0162
**employment_l6m**	**Yes**	1	0.1047	0.2144	0.2385	0.6253
**street_inc_l6m**	**Yes**	1	0.2318	0.2056	1.2705	0.2597
**ill_inc_l6m**	**Yes**	1	-0.2089	0.1986	1.1071	0.2927
**sexwork_inc_l6m**	**Yes**	1	0.3434	0.3029	1.2854	0.2569

Appendix 3. Details of the external validation data used in the treatment (exposure) misclassification bias analysis.

For the present study, we specified the sensitivity and specificity of self-reported current opioid agonist treatment (A_{it-1}; our focal exposure) based on external validation data from a prior study by Bouck et al.[8] In brief, Bouck et al. assessed the accuracy of participant-reported measures of current (and recent) opioid agonist treatment in a sample of 521 Toronto-based people who inject drugs (all aged 18 or older with injection drug use in the past six months).[8] The below table summarizes their results on the concurrent validity of self-reported current opioid agonist treatment relative to comprehensive prescription dispensation claims data.[8]

Parameter	Estimate	95% Confidence Interval
Sensitivity	84%	78% to 90%
Specificity	87%	83% to 91%

From the above table, we calculated the shape parameters (alpha [α] and beta [β]) to specify the beta distribution for both classification parameters (sensitivity and specificity). Explicitly, per classification parameter, we used the 95% confidence interval limits to calculate the standard deviation (SD = [upper limit – lower limit]/[2*1.96]). We then calculated the shape parameters as follows[9]:

$$\alpha = x\left(\frac{x(1-x)}{\mathrm{SD}^2} - 1\right)$$

$$\beta = (1-x) * \left(\frac{x(1-x)}{\mathrm{SD}^2} - 1\right)$$

Where x is the corresponding mean parameter value on the proportion scale, i.e., 0.84 for sensitivity and 0.87 for specificity.

Appendix 4. SAS code for treatment misclassification—exposure, A_{it-1} and covariate, A_{it-2}—probabilistic bias analyses.

Code for this analysis was adapted from open-access SAS code provided at the following website (https://sites.google.com/site/biasanalysis/) for a textbook chapter written by Fox and colleagues.[10]

```
*SA#2: Probabilistic QBA to correct for misclassification of exposure (At-1)
and covariate (At-2);

****** SAS CODE FOR RECORD LEVEL BIAS ANALYSIS: EXPOSURE MISCLASSIFICATION*;
* Created by:     Matt Fox
* Date created:   Sept 19, 2018
* Modified by ZB to fit weighted repeated measures outcome model
* Purpose:        Simple SAS code for individual record level PBA for
exposure (and covariate) misclassification
****************************************************************************;
proc datasets library=work kill;
run;
proc contents data=PhD_aim2.bl19_AAV_long_lag;
run; *1448 obs (t=0 to 5);
proc freq data=PhD_aim2.bl19_AAV_long_lag;
table t; /*at each time point (t range 0-4), lag_OAT_curr, OAT_curr, and
pIIA_p6m measurement*/
run;
proc freq data=PhD_aim2.bl19_AAV_long_lag;
where t ge 1;
table OAT_c_lb*lag_OAT_curr OAT_curr lag_OAT_curr*pIIA_p6m;
run;

*Added by ZB: Create temp dataset
      (creates 10000 copies/replicates of original dataset, with all copies
contained within single dataset - misc);
data misc;
set PhD_aim2.bl19_AAV_long_lag;
do iter=1 to 10000; output; end;
run; *1448 (1114+334)*10000 = 14480000 obs as expected;

* output summary dataset;
proc freq data=misc noprint; where iter = 1; tables
lag_OAT_curr*pIIA_p6m/out=test; format _all_; run;

* collapse to one observation for the entire dataset;
data test2; set test;
      retain ed_o ued_o eud_o ueud_o;
      if lag_OAT_curr = 1 and pIIA_p6m = 1 then ed_o = count;
      else if lag_OAT_curr = 1 and pIIA_p6m = 0 then eud_o = count;
      else if lag_OAT_curr = 0 and pIIA_p6m = 1 then ued_o = count;
      else if lag_OAT_curr = 0 and pIIA_p6m = 0 then ueud_o = count;
      if _n_ = 4 then output;
      keep ed_o ued_o eud_o ueud_o;
run;

* manipulate the data and Se and Sp to get PPVs and NPVs;
```

```
data test3; set test2;

      do  iter = 1 to 10000; * change to determine the number of simulations
you want to run;
      * for each iteration, generate sensitivity and specificity, change
values to your chosen distributions;

*use this code if you want non-differential misclassification;
      * sensitivity and specificity;
            se_D=rand('beta', 116.6, 22.2); /*mean = 84%, SD = 3.1%*/
            sp_D=rand('beta', 245.1, 36.6); /*mean = 87%, SD = 2.0%*/
      * for ND misclassification, set se_UD = se_D and  sp_UD = sp_D;
            se_UD = se_D;
            sp_UD = sp_D;
      * calculate expected "truth" based on sens and spec;
            ed_t=(ed_o-(1-sp_d)*(ed_o+ued_o))/(se_d-(1-sp_d));
            ued_t=(ed_o+ued_o)-ed_t;
            eud_t=(eud_o-(1-sp_ud)*(eud_o+ueud_o))/(se_ud-(1-sp_ud));
            ueud_t=(eud_o+ueud_o)-eud_t;
      * expected misclassification adjusted;
            syst_exp_adj = (ed_t /eud_t) / (ued_t /ueud_t);
      * count only if all cells are positive;
            if ed_t > 0 and ued_t > 0 and eud_t > 0 and ueud_t > 0 then do;

            * sample expected prevalence;
                  prev_e_d = rand('beta', ed_t, ued_t);
                  prev_e_ud = rand('beta', eud_t, ueud_t);
            * calculate PPV and NPV;
                  PPV_d=(se_d* prev_e_d)/((se_d* prev_e_d)+((1-sp_d)*(1-
prev_e_d)));
                  NPV_d=(sp_d * (1-prev_e_d))/(((1-se_d) *
prev_e_d)+(sp_d*(1-prev_e_d)));
                  PPV_ud=(se_ud* prev_e_ud)/((se_ud* prev_e_ud)+((1-
sp_ud)*(1-prev_e_ud)));
                  NPV_ud=(sp_ud * (1-prev_e_ud))/(((1-se_ud) *
prev_e_ud)+(sp_ud*(1-prev_e_ud)));
            end;
            else do;
            * if iteration produces negative cells, set everything to 0;
                  prev_e_d = .; prev_e_ud = .;
                  PPV_d=.; NPV_d=.; PPV_ud=.; NPV_ud=.;
            end;
            output;
      end;
run;

/*Added*/
proc means data=test3 noprint; by iter; var se_D sp_D PPV_d PPV_ud NPV_d
NPV_ud prev_e_d prev_e_ud;
output out=sstemp mean=se_D sp_D PPV_d PPV_ud NPV_d NPV_ud prev_e_d
prev_e_ud; run;
data selectedAparms;
set sstemp;
drop _:;
run;
*describe distribution of selected parm values across all iterations (none
failed);
proc means data=selectedAparms n min mean stddev max;
```

```
var se_D sp_D PPV_d PPV_ud NPV_d NPV_ud prev_e_d prev_e_ud;
run;
proc univariate data=selectedAparms;
var se_D;
histogram/beta(alpha=116.6, beta=22.2);
label se_D="Sensitivity";
run;
proc univariate data=selectedAparms;
var sp_D;
histogram/beta(alpha=245.1, beta=36.6);
label sp_D="Specificity";
run;
proc univariate data=selectedAparms;
var PPV_d;
histogram;
label PPV_d="PPV for Y=1";
run;
proc univariate data=selectedAparms;
var NPV_d;
histogram;
label NPV_d="NPV for Y=1";
run;
proc univariate data=selectedAparms;
var PPV_ud;
histogram;
label PPV_ud="PPV for Y=0";
run;
proc univariate data=selectedAparms;
var NPV_ud;
histogram;
label NPV_ud="NPV for Y=0";
run;

* merge PPV and NPV back into the main dataset "misc";
proc contents data=misc; run;
proc contents data=test3; run; *10000 obs, 1 per iter;
proc sort data=misc; by iter id t; run;
proc sort data=test3; by iter; run;

* use PPV and NPV to simulate adjusted data;
data misc2; merge misc test3; by iter;
      if lag_OAT_curr = 1 and pIIA_p6m = 1 and PPV_d ne . then
lag_OAT_curr_t = rand('Bernoulli',PPV_d); *lag_OAT_curr_t = simulated
corrected exposure response;
      else if lag_OAT_curr = 0 and pIIA_p6m = 1 and NPV_d ne . then
lag_OAT_curr_t = rand('Bernoulli',1-NPV_d);
      else if lag_OAT_curr = 1 and pIIA_p6m = 0 and PPV_ud ne . then
lag_OAT_curr_t = rand('Bernoulli',PPV_ud);
      else if lag_OAT_curr = 0 and pIIA_p6m = 0 and NPV_ud ne . then
lag_OAT_curr_t = rand('Bernoulli',1-NPV_ud);
run;

*ZB added: Update lag2_OAT_curr values based on lag(lag_OAT_curr_t) ->
correcting covariate (At-2);
proc sort data=misc2; by iter id t; run;
/*Note: update covariate based on lag of exposure vs conducting another
Bernoulli simulation. This ensures that treatment history is
```

```
consistent over time (i.e., lag2_OAT_curr_t at visit t = lag_OAT_curr_t at
visit t-1)*/
data misc3;
set misc2;
by iter id;
lag2_OAT_curr_t=lag(lag_OAT_curr_t);
if first.id then lag2_OAT_curr_t=.; /*where t=0, A(-2) not measured so set
to missing*/
run;

*conventional result, unweighted (should mirror result from step 14);
ods select none;
proc genmod data=misc3 desc;
where iter=1 and t in (1,2,3,4,5);
class id t(ref="1") lag_OAT_curr(ref="0") lag2_OAT_curr(ref="0")/param=ref;
model pIIA_p6m /*Yt*/ = t lag_OAT_curr /*At-1*/ lag2_OAT_curr /*At-
2*//dist=bin link=log;
repeated subject=id/within=t corrw type=un;
ods output GEEEmpPEst=conv_IPTC_parms_uw;
run; *estimates identical (ln(RR)=-0.6227, with SE(ln[RR])=0.3988 from Table
3;
ods select all;
proc print data=conv_IPTC_parms_uw;
run;

*(a) fit unweighted outcome model;
ods select none;
proc genmod data=misc3 desc; by iter;
where t in (1,2,3,4,5);
class id t(ref="1") lag_OAT_curr_t(ref="0")
lag2_OAT_curr/*_t*/(ref="0")/param=ref;
model pIIA_p6m /*Yt*/ = t lag_OAT_curr_t /*At-1*/ lag2_OAT_curr /*At-
2*//dist=bin link=log;
repeated subject=id/within=t corrw type=un;
ods output GEEEmpPEst=SA2_parms_uw;
run;
ods select all;
proc contents data=SA2_parms_uw;
run;

proc univariate data=SA2_parms_uw;
where parm="lag_OAT_curr_t";
var estimate;
run;

/*calculate median RR and 95% SI (random + systematic error)*/
data SA2_parms_exp_uw;
set SA2_parms_uw;
where parm="lag_OAT_curr_t";
      e_syst=estimate;
      stderr_syst=stderr;
      e_conv=-0.6227;
      stderr_conv=0.3988;
      RR_rand=exp(e_conv - rand('normal')*stderr_conv);
      RR_tot=exp(e_syst - rand('normal')*stderr_syst); /*Fox (2021) approach
to incorporating random error*/
      RR_syst=exp(e_syst);
run;
```

```
* print out the 2.5th-97.5th percentile;
proc univariate data=SA2_parms_exp_uw;
var RR_rand RR_syst RR_tot;
output out=SA2_parms_exp_uw2 pctlpts=2.5 50 97.5 pctlpre= RAND_ SYST_
TOTAL_;
run;

* manipulate dataset to make readable;
data niceprint; set SA2_parms_exp_uw2;
      analysis="Random error     ";result = RAND_50; result_2_5 =RAND_2_5;
result_97_5 =RAND_97_5; width = result_97_5/result_2_5;output;
      analysis="Systematic error";result = SYST_50; result_2_5 =SYST_2_5;
result_97_5 =SYST_97_5; width = result_97_5/result_2_5;output;
      analysis="Total error      ";result = TOTAL_50; result_2_5 =TOTAL_2_5;
result_97_5 =TOTAL_97_5; width = result_97_5/result_2_5;output;
run;

* print results;
proc print data=niceprint label;
      var analysis result result_2_5 result_97_5 width;
      label result="Median" result_2_5="2.5th pctl" result_97_5="97.5th
pctl" width = "Width of interval";
run;

/*free up resources by deleting temp datasets that are no longer needed*/
proc delete library=work data=SA2_parms_exp_uw2 SA2_parms_exp_uw
      SA2_parms_uw conv_IPTC_parms_uw
      selectedAparms sstemp misc misc2 test test2 test3;
run;
****;

*(b) simultaneous correction for exposure (At-1) and covariate term (At-2)
capturing treatment over past two visits;
ods select none;
proc genmod data=misc3 desc; by iter;
where t in (1,2,3,4,5);
class id t(ref="1") lag_OAT_curr_t(ref="0")
lag2_OAT_curr_t(ref="0")/param=ref;
model pIIA_p6m /*Yt*/ = t lag_OAT_curr_t /*At-1*/ lag2_OAT_curr_t /*At-
2*//dist=bin link=log;
repeated subject=id/within=t corrw type=un;
ods output GEEEmpPEst=SA2_AX_parms_uw;
run;
ods select all;
proc contents data=SA2_AX_parms_uw;
run;

proc univariate data=SA2_AX_parms_uw;
where parm="lag_OAT_curr_t";
var estimate;
run;

/*calculate median RR and 95% SI (random + systematic error)*/
data SA2_AX_parms_exp_uw;
set SA2_AX_parms_uw;
where parm="lag_OAT_curr_t";
      e_syst=estimate;
```

```
      stderr_syst=stderr;
      e_conv=-0.6227;
      stderr_conv=0.3988;
      RR_rand=exp(e_conv - rand('normal')*stderr_conv);
      RR_tot=exp(e_syst - rand('normal')*stderr_syst); /*Fox (2021) approach
to incorporating random error*/
      RR_syst=exp(e_syst);
run;

* print out the 2.5th-97.5th percentile;
proc univariate data=SA2_AX_parms_exp_uw;
var RR_rand RR_syst RR_tot;
output out=SA2_AX_parms_exp_uw2 pctlpts=2.5 50 97.5 pctlpre= RAND_ SYST_
TOTAL_;
run;

* manipulate dataset to make readable;
data niceprint2; set SA2_AX_parms_exp_uw2;
      analysis="Random error    ";result = RAND_50; result_2_5 =RAND_2_5;
result_97_5 =RAND_97_5; width = result_97_5/result_2_5;output;
      analysis="Systematic error";result = SYST_50; result_2_5 =SYST_2_5;
result_97_5 =SYST_97_5; width = result_97_5/result_2_5;output;
      analysis="Total error      ";result = TOTAL_50; result_2_5 =TOTAL_2_5;
result_97_5 =TOTAL_97_5; width = result_97_5/result_2_5;output;
run;

* print results;
proc print data=niceprint2 label;
      var analysis result result_2_5 result_97_5 width;
      label result="Median" result_2_5="2.5th pctl" result_97_5="97.5th
pctl" width = "Width of interval";
run;
**************************************************************************;
```

Appendix Figure 1. Distributions for sensitivity and specificity of exposure (current first-line opioid agonist treatment at visit *t*-1) based on 10,000 iterations. *Notes:* Curved line outlines specified beta distributions (α, β) for each parameter. [a] Lagged to prior visit. [b] Exposure misclassification non-differential with respect to outcome (recent injection initiation assistance provision at visit *t*).

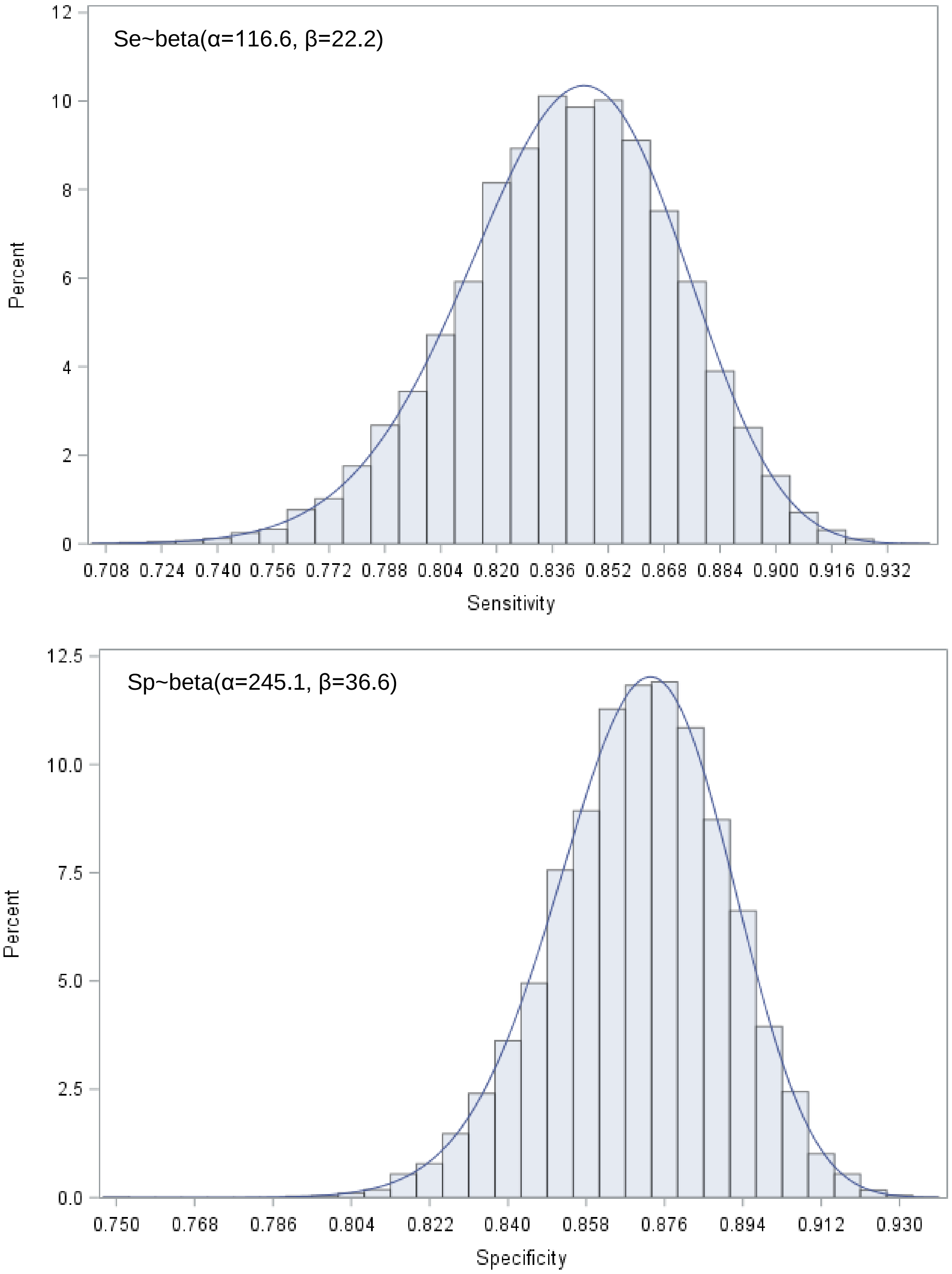

Appendix 5. SAS code for outcome misclassification probabilistic bias analysis.

Code for this analysis was adapted from open-access SAS code provided at the following website (https://sites.google.com/site/biasanalysis/) for a textbook chapter written by Fox and colleagues.[10]

```
*SA#3: Probabilistic QBA to correct for differential misclassification of
outcome (Yt) by exposure (At-1);

proc datasets library=work kill;
run;
proc contents data=PhD_aim2.bl19_AAV_long_lag;
run;

*Added by ZB: Create temp dataset
      (creates 10000 copies/replicates of original dataset, with all copies
contained within single dataset - misc);
data misc;
set PhD_aim2.bl19_AAV_long_lag;
do iter=1 to 10000; output; end;
run; *1448 (1114+334)*10000 = 14480000 obs as expected;

* output summary dataset;
proc freq data=misc noprint; where iter = 1; tables
pIIA_p6m*lag_OAT_curr/out=test; format _all_; run;

* collapse to one observation for the entire dataset;
data test2; set test;
      retain ed_o ued_o eud_o ueud_o;
      if pIIA_p6m = 1 and lag_OAT_curr = 1 then ed_o = count; /*for sake of
repurposing code from A m/c QBA, letting Y here be "exposure" and A be
“outcome”*/
      else if pIIA_p6m = 1 and lag_OAT_curr = 0 then eud_o = count;
      else if pIIA_p6m = 0 and lag_OAT_curr = 1 then ued_o = count;
      else if pIIA_p6m = 0 and lag_OAT_curr = 0 then ueud_o = count;
      if _n_ = 4 then output;
      keep ed_o ued_o eud_o ueud_o;
run;

* manipulate the data and Se and Sp to get PPVs and NPVs;
data test3; set test2;

      do  iter = 1 to 10000; * change to determine the number of simulations
you want to run;
      * for each iteration, generate sensitivity and specificity, change
values to your chosen distributions;

/*differential m/c of outcome by exposure status*/
            se_D=rand('beta',33.7,9.5); /*on current OAT*/
            se_UD=rand('beta',40.6,16.6); /*not on current OAT*/
            sp_D=rand('beta',788.5,0.3); /*on current OAT*/
            sp_UD=rand('beta',558.4,0.4); /*not on current OAT*/

      * calculate expected "truth" based on sens and spec;
            ed_t=(ed_o-(1-sp_d)*(ed_o+ued_o))/(se_d-(1-sp_d));
            ued_t=(ed_o+ued_o)-ed_t;
            eud_t=(eud_o-(1-sp_ud)*(eud_o+ueud_o))/(se_ud-(1-sp_ud));
```

```
            ueud_t=(eud_o+ueud_o)-eud_t;

      * count only if all cells are positive;
            if ed_t > 0 and ued_t > 0 and eud_t > 0 and ueud_t > 0 then do;

            * sample expected prevalence;
                  prev_e_d = rand('beta', ed_t, ued_t);
                  prev_e_ud = rand('beta', eud_t, ueud_t);
            * calculate PPV and NPV;
                  PPV_d=(se_d* prev_e_d)/((se_d* prev_e_d)+((1-sp_d)*(1-
prev_e_d)));
                  NPV_d=(sp_d * (1-prev_e_d))/(((1-se_d) *
prev_e_d)+(sp_d*(1-prev_e_d)));
                  PPV_ud=(se_ud* prev_e_ud)/((se_ud* prev_e_ud)+((1-
sp_ud)*(1-prev_e_ud)));
                  NPV_ud=(sp_ud * (1-prev_e_ud))/(((1-se_ud) *
prev_e_ud)+(sp_ud*(1-prev_e_ud)));
            end;
            else do;
            * if iteration produces negative cells, set everything to
missing;
                  prev_e_d = .; prev_e_ud = .;
                  PPV_d=.; NPV_d=.; PPV_ud=.; NPV_ud=.;
            end;
            output;
      end;
run;

/*Added*/
proc means data=test3 noprint; by iter; var se_D se_UD sp_D sp_UD;
output out=sstemp mean=se_D se_UD sp_D sp_UD; run;
data selectedYparms;
set sstemp;
drop _:;
run;
*describe distribution of selected parm values across all iterations (none
failed);
proc means data=selectedYparms n min mean stddev max;
var se_D se_UD sp_D sp_UD;
run;
proc univariate data=selectedYparms;
var se_D;
histogram/beta(alpha=33.7, beta=9.5);
label se_D="Sensitivity";
title "Current first-line OAT (At-1)=Yes";
run;
proc univariate data=selectedYparms;
var se_UD;
histogram/beta(alpha=40.6, beta=16.6);
label se_UD="Sensitivity";
title "Current first-line OAT (At-1)=No";
run;
proc univariate data=selectedYparms;
var sp_D;
histogram/beta(alpha=788.5, beta=0.3);
label sp_D="Specificity";
title "Current first-line OAT (At-1)=Yes";
run;
```

```
proc univariate data=selectedYparms;
var sp_UD;
histogram/beta(alpha=558.4, beta=0.4);
label sp_UD="Specificity";
title "Current first-line OAT (At-1)=No";
run;

* merge PPV and NPV back into the main dataset misc;
proc contents data=misc;
run;
proc contents data=test3;
run;
data misc_reduced;
set misc;
keep iter id t pIIA_p6m lag_OAT_curr lag2_OAT_curr;
run;

proc sort data=misc_reduced; by iter; run;
proc sort data=test3; by iter; run;

* use PPV and NPV to simulate adjusted data;
data misc2; merge misc_reduced test3; by iter;
      if pIIA_p6m = 1 and lag_OAT_curr = 1 and PPV_d ne . then pIIA_p6m_t =
rand('Bernoulli',PPV_d);
      else if pIIA_p6m = 0 and lag_OAT_curr = 1 and NPV_d ne . then
pIIA_p6m_t = rand('Bernoulli',1-NPV_d);
      else if pIIA_p6m = 1 and lag_OAT_curr = 0 and PPV_ud ne . then
pIIA_p6m_t = rand('Bernoulli',PPV_ud);
      else if pIIA_p6m = 0 and lag_OAT_curr = 0 and NPV_ud ne . then
pIIA_p6m_t = rand('Bernoulli',1-NPV_ud);
run;
proc contents data=misc2;
run;

*now fit unweighted outcome model;
ods select GEEEmpPEst;
proc genmod data=misc2 desc;
by iter;
where t in (1,2,3,4,5);
class id t(ref="1") lag_OAT_curr(ref="0") lag2_OAT_curr(ref="0")/param=ref;
model pIIA_p6m_t /*Yt after bias-corrections*/ = t lag_OAT_curr /*At-1*/
lag2_OAT_curr /*At-2*//dist=bin link=log;
repeated subject=id/within=t corrw type=un;
ods output GEEEmpPEst=SA3_IPTC_parms_uw;
run;

/*calculate median RR and 95% SI (random + systematic error)*/
data SA3_AX_parms_uw;
set SA3_IPTC_parms_uw;
where parm="lag_OAT_curr";
      e_syst=estimate;
      stderr_syst=stderr;
      e_conv=-0.6227;
      stderr_conv=0.3988;
      RR_rand=exp(e_conv - rand('normal')*stderr_conv);
      RR_tot=exp(e_syst - rand('normal')*stderr_syst); /*Fox (2021) approach
to incorporating random error*/
      RR_syst=exp(e_syst);
```

```
run;

* print out the 2.5th-97.5th percentile;
proc univariate data=SA3_AX_parms_uw;
var RR_rand RR_syst RR_tot;
output out=SA3_AX_parms_uw2 pctlpts=2.5 50 97.5 pctlpre= RAND_ SYST_ TOTAL_;
run;

* manipulate dataset to make readable;
data niceprint3; set SA3_AX_parms_uw2;
      analysis="Random error    ";result = RAND_50; result_2_5 =RAND_2_5;
result_97_5 =RAND_97_5; width = result_97_5/result_2_5;output;
      analysis="Systematic error";result = SYST_50; result_2_5 =SYST_2_5;
result_97_5 =SYST_97_5; width = result_97_5/result_2_5;output;
      analysis="Total error      ";result = TOTAL_50; result_2_5 =TOTAL_2_5;
result_97_5 =TOTAL_97_5; width = result_97_5/result_2_5;output;
run;

* print results;
proc print data=niceprint3 label;
      var analysis result result_2_5 result_97_5 width;
      label result="Median" result_2_5="2.5th pctl" result_97_5="97.5th
pctl" width = "Width of interval";
run;

/*free up resources by deleting temp datasets that are no longer needed*/
proc delete library=work data=SA3_AX_parms_uw SA3_IPTC_parms_uw
SA3_AX_parms_uw2 niceprint3;
run;
*******************************************************************;
```

Appendix Figure 2. Distributions for sensitivity and specificity of outcome (recent injection initiation assistance provision at visit *t*) by reported exposure status (current first-line opioid agonist treatment [OAT] at visit *t*-1) based on 10,000 iterations. *Notes:* Curved line outlines specified beta distributions (α, β) for each parameter. [a] Lagged to prior visit.

(a) Current first-line OAT[a] = Yes **(b) Current first-line OAT[a] = No**

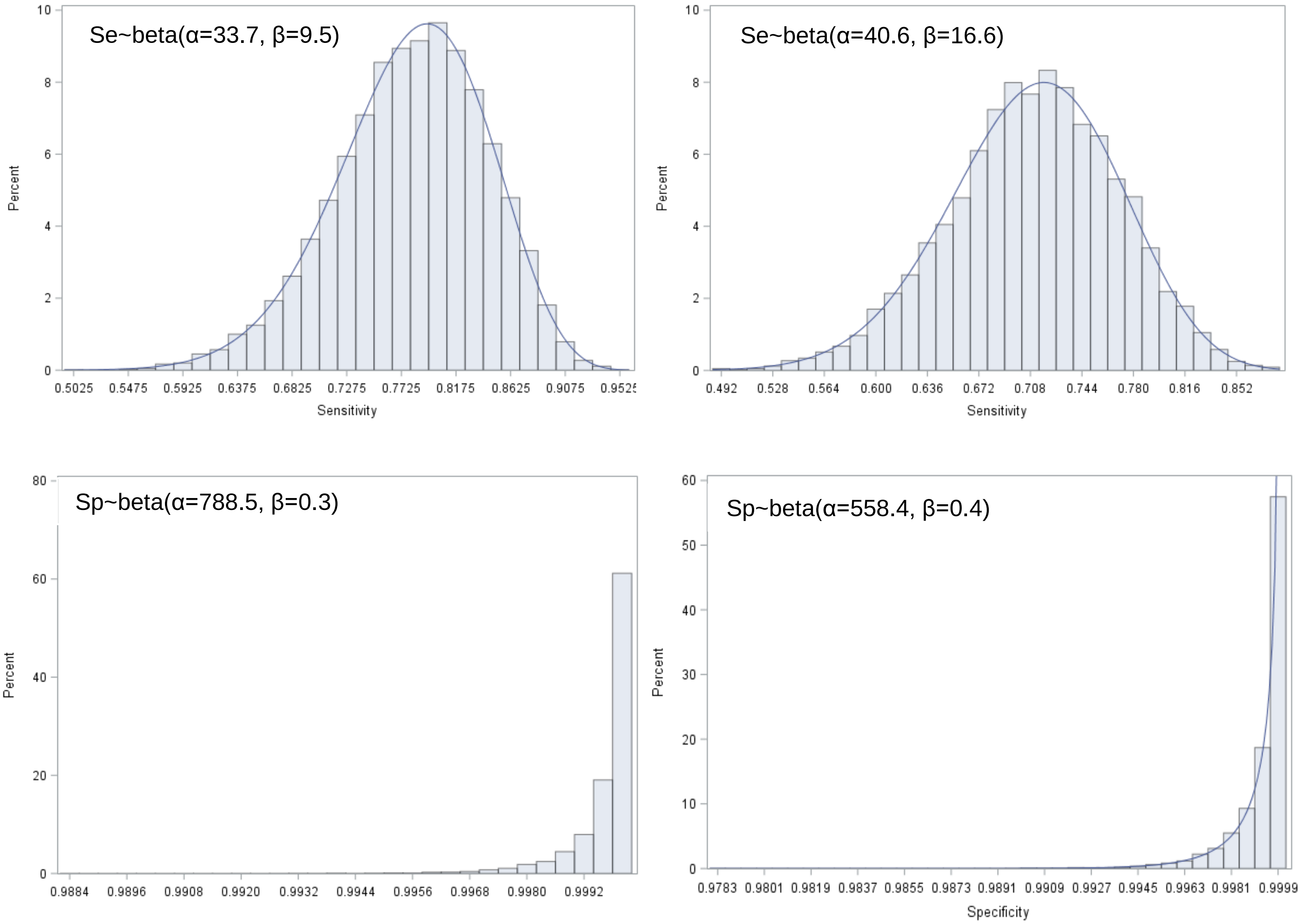

Appendix Table 1. Alternative 95% CI calculated using non-parametric percentile bootstrapping with individual participants as cluster sampling units.

Model specifications	# of iterations that converged	Effect of current OAT at visit *t*-1 RR	95% CI[a]	% change in width versus robust 95% CI
Unweighted	1392	0.54	0.23 to 1.24	+10%
IPC weighted	1381	0.56	0.25 to 1.17	-6%
IPT weighted	1329	0.47	0.21 to 1.09	+5%
IPTC weighted	1284	0.50	0.23 to 1.14	+3%

Notes: RR = Relative risk; IPC = inverse-probability-of-censoring; IPT = inverse-probability-of-treatment; IPTC = inverse-probability-of-treatment-and-censoring; CI = confidence interval.
[a] Constructed using percentile bootstrapping with 1500 bootstrapped samples and participants as the cluster sampling unit.
[b] Calculated as: [(Width of bootstrapped CI – width of robust CI from **Table 3**)/(Width of robust CI from **Table 3**)]*100. Therefore, a positive value indicates that the bootstrapped CI is wider than the robust CI whereas a negative value indicates that the bootstrapped CI is narrower than the robust CI.

REFERENCES

1. Oviedo-Joekes E, Guh D, Brissette S, Marchand K, MacDonald S, Lock K, et al. Hydromorphone Compared With Diacetylmorphine for Long-term Opioid Dependence: A Randomized Clinical Trial. JAMA Psychiatry. 2016 May 1;73(5):447.

2. Study to Assess Long-term Opioid Maintenance Effectiveness (SALOME) [Internet]. [cited 2022 Apr 12]. Available from: https://www.providencehealthcare.org/salome/

3. British Columbia Centre on Substance Use. A Guideline for the Clinical Management of Opioid Use Disorder [Internet]. 2017 [cited 2022 Apr 12]. Available from: https://www.bccsu.ca/wp-content/uploads/2017/06/BC-OUD-Guidelines_June2017.pdf

4. Hernán MA, Brumback BA, Robins JM. Estimating the causal effect of zidovudine on CD4 count with a marginal structural model for repeated measures. Statist Med. 2002 Jun 30;21(12):1689–709.

5. Cole SR, Hernán MA. Constructing Inverse Probability Weights for Marginal Structural Models. American Journal of Epidemiology. 2008 Jul 15;168(6):656–64.

6. Moodie EEM, Delaney JAC, Lefebvre G, Platt RW. Missing Confounding Data in Marginal Structural Models: A Comparison of Inverse Probability Weighting and Multiple Imputation. The International Journal of Biostatistics [Internet]. 2008 Jan 14 [cited 2021 Jun 10];4(1). Available from: https://www.degruyter.com/document/doi/10.2202/1557-4679.1106/html

7. Hernán MA, Robins JM. Causal Inference: What If. 2020;311.

8. Bouck Z, Tricco AC, Rosella LC, Ling V, Gomes T, Tadrous M, et al. Validation of self-reported opioid agonist treatment among people who inject drugs using prescription dispensation records. Epidemiology. 2022 Mar;33(2).

9. Lash TL, Fox MP, Fink AK. Applying Quantitative Bias Analysis to Epidemiologic Data. Springer; 2009.

10. Fox MP, MacLehose RF, Lash TL. Probabilistic Bias Analysis for Simulation of Record-Level Data. In: Fox MP, MacLehose RF, Lash TL, editors. Applying Quantitative Bias Analysis to Epidemiologic Data [Internet]. Cham: Springer International Publishing; 2021. p. 291–327. Available from: https://doi.org/10.1007/978-3-030-82673-4_9
